# Heterogeneity of treatment preferences in the absence of guideline recommendations – a case vignette study in colorectal cancer tumor boards in Germany, Austria and Switzerland

**DOI:** 10.1186/s12876-025-04183-5

**Published:** 2025-10-07

**Authors:** Johannes Soff, Stefan Rolf Benz, Christoph Kowalski, Judith Hansinger, Michael Gerken, Daniel Maier, Soo-Zin Kim-Wanner, Jacqueline Witzmann, Anke Reinacher-Schick, Christian Peter Pox, Kees Kleihues-van Tol, Bianca Franke, Olaf Schoffer, Jochen Schmitt, Monika Klinkhammer-Schalke, Vinzenz Völkel

**Affiliations:** 1 Department of Health Services Research, German Cancer Society, Kuno-Fischer-Strasse 8, 14057 Berlin, Germany; 2 Association of DKG-Certified German Colorectal Cancer Centers, Goernestrasse 30, 20249 Hamburg, Germany; 3 Department for Abdominal and Pediatric Surgery, Klinikverbund-Suedwest, Klinken Böblingen, Böblingen, Germany; 4 Tumor Center Regensburg, Center of Quality Management and Health Services Research, University of Regensburg, Am Biopark 9, 93053 Regensburg, Germany; 5 Bavarian Cancer Research Center (BZKF), Regensburg, Germany; 6 Bavarian Cancer Registry, Bavarian Health and Food Safety Authority, Schweinauer Hauptstraße 80, 90441 Nuremberg, Germany; 7 Faculty of Medicine, Institute for Digital Medicine and Clinical Data Science, Goethe University Frankfurt, Frankfurt Am Main, Germany; 8 German Cancer Consortium (DKTK), Partner Site Frankfurt/Mainz, a partnership between DKFZ and the University Hospital Frankfurt, Frankfurt Am Main, Germany; 9 Hessian Office for Health and Care, Hessian Cancer Registry, Lurgiallee 10, 60439 Frankfurt Am Main, Germany; 10 Association of German Tumor Centers (ADT), Kuno-Fischer-Strasse 8, 14057 Berlin, Germany; 11 Hämatologie, Onkologie und Palliativmedizin, Katholisches Klinikum, Ruhr-Universität Bochum, Gudrunstrasse 56, 44791 Bochum, Germany; 12 Medizinische Klinik, Joseph-Stift Bremen, Schwachhauser Heerstrasse 54, 28209 Bremen, Germany; 13 Center for Evidence-Based Healthcare (ZEGV), Faculty of Medicine, University Hospital Carl Gustav Carus and Carl Gustav Carus, TU Dresden, Fetscherstr. 74, 01307 Dresden, Germany

**Keywords:** Treatment preference, Oncology, Guidelines, Certified cancer center, Case vignettes, Survey study

## Abstract

**Background:**

For the treatment of colorectal cancer, the S3-Guideline of the German Guideline Program in Oncology supports clinical decision-making. Centers certified by the German Cancer Society are required to implement the guideline recommendations as comprehensively as possible. When guidelines provide insufficient or ambiguous evidence, heterogeneity of treatment preferences is likely to emerge across individual centers. The aim of this study is to describe the preferences of the centers’ tumor boards for treatment decisions when clear, evidence-based guideline recommendations are lacking.

**Methods:**

To investigate the tumor boards’ preferences for different treatment options, an anonymous online survey was conducted among 314 certified colorectal cancer centers. The survey included seven visceral oncological and nine visceral surgical case vignettes. Centers were asked to discuss the vignettes in the tumor board or, alternatively, delegate them to an appropriate physician representatively speaking for the tumor board. The responses were analyzed descriptively and normalized entropy estimates (NE) were calculated for each vignette.

**Results:**

A total of 123 centers (39%) responded to the survey. For oncological cases without clear guideline recommendations, substantial heterogeneity (NE: 0.39–0.71) in treatment preferences was observed. For instance, opinions varied widely for UICC II pT4a colon cancer. In this situation, 28% of centers would prefer fluoropyrimidine monotherapy, 39% oxaliplatin-based combination therapy and 33% no adjuvant chemotherapy at all. Surgical vignettes showed a preference for laparoscopic and robotic approaches, with variations based on tumor location (NE: 0.46–0.67). Importantly, in case of a clear evidence-based guideline recommendation, treatment preferences did not differ considerably between hospital sites.

**Conclusions:**

In prototypical case vignettes without evidence-based guideline recommendations, pronounced heterogeneity of treatment preferences between centers was found. Reconstructing these treatment preferences can contribute to enhancing the quality of evidence derived from observational studies. This seems especially important in the context of clinical questions that cannot be assessed in randomized trials; clinical routine data represent an adequate resource for evidence generation in these scenarios.

**Trial registration:**

After conducting this study, the main study was registered in the German Clinical Trials Register (DRKS) on October 4, 2024 under study No. DRKS00034650.

**Supplementary Information:**

The online version contains supplementary material available at 10.1186/s12876-025-04183-5.

## Introduction

Colorectal cancer is the third most common type of cancer in men and the second most common cancer in women both worldwide with approximately 1.8 million new cases per year [1] and in Germany with approximately 54,000 new cases per year [2]. Medical treatment decisions, especially in advanced colorectal cancer, are often complex and survival time alone may not be the single most important outcome, considering the patients’ life expectancy and possible limitations in function and quality of life due to the side effects of treatment. In Germany, the S3-Guideline “Colorectal Cancer” of the German Guideline Program in Oncology serves as a basis for supporting clinical decisions [3]. S3-Guidelines are both evidence- and consensus-based and comprise a systematic search, selection and evaluation of scientific evidence. The recommendations of the guideline also provide the basis of the certification criteria of the German Cancer Society [4]. Certified colorectal cancer centers are required to implement the guideline recommendations as comprehensively as possible. All centers must provide access to the appropriate therapies by ensuring the availability of medication and clinical expertise. However, in cases where the guideline does not provide a clear recommendation, different treatment preferences are likely to develop across individual centers [5, 6]. While evidence on the development and impact of treatment preferences in colorectal cancer is scarce, organizational theories such as new institutionalism provide possible hypotheses [7]. According to this theory, persistent and collectively shared management and treatment preferences arise in hierarchically structured organizations like hospitals. The preferences for a treatment reflect the medical teams’ evaluation of the relative benefits, harms, costs and inconveniences in comparison with the given alternatives for an individual patient [8]. This assumed institutional treatment preference influences the individual preferences of the treating physicians. Regardless of the availability of evidence, the physicians’ treatment recommendation is central to the treatment decision [9] and in certain cases trumps the patients’ preference [10, 11]. In cancer treatment, this refers, for example, to different health states after chemotherapy that are evaluated differently by various practitioners [12, 13]. In similar medical decision-making processes in oncology, factors not associated with the indication for treatment, such as the physicians’ personalities, seem to contribute to the treatment choice [13]. For rectal cancer, Harrison et al. [14], for example, showed that surgeons and oncologists had differing preferences for adjuvant treatment options.

The Cancer Research Data Center (onkoFDZ) project [15] aims to approximate the institutions‘ treatment preferences based on their therapy allocation behavior in routine practice data for cases in which randomized controlled trials cannot be applied or are avoided due to practical or ethical reasons [16]. Underlying institutional treatment preferences cannot be directly observed in the routine data itself and the extent and heterogeneity of these mechanisms are poorly studied [17]. In categorical systems, such as distinct therapy decisions, heterogeneity describes the degree to which therapy decisions diverge from a state of perfect conformity [18]. It remains unclear whether the observed differences in prescribing behavior between institutions truly reflect differences in preference or merely result from unmeasured differences in their patient populations [19]. Consequently, this potentially useful role of treatment preferences as an instrument variable only holds if the assumption of heterogeneous treatment preferences can be shown for the clinical use cases of interest [20].

The aim of the study is therefore to describe the preferences of tumor boards from colorectal cancer centers for treatment decisions without clear, evidence-based guideline recommendations, as a possible basis for evidence generation in routine practice data.

## Materials and methods

### Study design and setting

To investigate the tumor boards’ preferences for different treatment options, we conducted a cross-sectional case vignette study [21, 22] within the onkoFDZ project using descriptions of specific risk factor or treatment constellations at the patient level. As no case vignette-specific reporting guideline is available yet, we applied the Strengthening the Reporting of Observational Studies in epidemiology (STROBE) guideline for cross-sectional studies [23] and the Consensus-Based Checklist for Reporting of Survey Studies (CROSS) [24].

We included all colorectal cancer centers certified in accordance with the requirements of the German Cancer Society at the time of the study, which are located in Germany, Austria, or Switzerland [25]. In order to meet the requirements in 2023, centers had to have a minimum annual caseload of 30 operative patients with primary colon cancer and 20 operative patients with primary rectum cancer. Moreover, centers need to make sure that every case is reviewed in a multidisciplinary tumor board meeting that discusses possible treatment options before going into the participatory decision-making process together with the patient. According to the certification requirements, the tumor board must always consist of specialists from visceral surgery, gastroenterology, radiotherapy, hematology or oncology, pathology and radiology.

### Case vignette development

The case vignettes were developed through a multi-step process. Initially, two clinical physicians created the case vignettes for the study based on guideline recommendations with grade 0 (“Recommendation open”) or B (“should/should not Recommendation “) which rely on limited evidence (Evidence Level According to Oxford (Version 2009) ≤ 3b) and control vignettes with recommendation grade A and existing evidence from a randomized controlled trial with narrow confidence interval (Evidence Level According to Oxford (Version 2009) 1b). Two head physicians from certified centers then refined the vignettes. One specialized in visceral surgery and hematology, and the other specialized in internal oncology. An interdisciplinary research team reviewed and implemented the proposed changes. Disagreements between the researchers were discussed, taking particular account of the clinical expertise of the specialists from the certified centers. The survey was then pre-tested in one tumor board meeting and finalized by a health services researcher. During the pre-test, terms in the description of the institution and the introductory text were clarified. The vignettes were perceived as intelligible and unambiguous. The final survey included seven case vignettes with visceral oncological questions regarding perioperative therapy, as well as nine case vignettes on visceral surgical questions. The possible answers of visceral oncological questions were limited to single-choice answers of various cytostatics or no chemotherapy. Two of these visceral oncological case vignettes were used as control cases to ensure that the response behavior was consistent in the presence of reliable evidence. To further differentiate the control cases, one of the two cases was based on a guideline recommendation for which more recent study evidence was available [26]. An overview of the visceral oncological case vignettes and the corresponding guideline recommendations can be found in Table 1. The visceral surgical questions addressed the localization-dependent choice of the surgical approach for the case of a patient with a colorectal tumor stage III (cT3 N1) according to the Union for International Cancer Control (UICC), a Body Mass Index (BMI) of 25 and no previous surgeries. The single-choice options were: laparoscopic, robotic, natural orifice transluminal endoscopic surgery (NOTES) and open surgery. To exploratively investigate whether institutional attributes of the centers affect the profiles of the treatment preferences, basic center information like the caseload of colon and rectum cancer patients and the teaching status of the center were surveyed.

**Table 1:** Case vignettes description

Case Number	Case description	Possible answers	Guideline Recommendation of the S3-Guideline [Number of Recommendation]^a^	Grades of Recommendation/Level of Evidence^b^
**Visceral oncological case vignettes on adjuvant chemotherapy (open recommendations)**				
#1	Colon cancer UICC Stage III pT3 N1 (2/25) L0 V0 Pn0 R0, ECOG 0, Age 81	No adjuvant chemotherapy; Adjuvant chemotherapy with fluoropyrimidine monotherapy (oral/IV); Adjuvant chemotherapy with oxaliplatin-based combination therapy; Adjuvant chemotherapy with another protocol	Adjuvant therapy should not be omitted solely for reasons of age. However, there is insufficient evidence to support the application of adjuvant chemotherapy in patients aged over 75 years. [8.1.]	B/NA (only expert consensus)
#2	Colon cancer UICC Stage II pT3 N0 (0/25) L0 V0 Pn0 G2 R0, MSS, ECOG 0, Age 73	No adjuvant chemotherapy; Adjuvant chemotherapy with fluoropyrimidine monotherapy (oral/IV); Adjuvant chemotherapy with oxaliplatin-based combination therapy; Adjuvant chemotherapy with another protocol	For patients with curatively resected stage II colon cancer, an adjuvant chemotherapy can be performed. [8.5.]	0/1b
#3	Colon cancer UICC Stage II pT4a N0 (0/25) L0 V0 Pn0 G2 R0, MSS, ECOG 0, Age 68	No adjuvant chemotherapy; Adjuvant chemotherapy with fluoropyrimidine monotherapy (oral/IV); Adjuvant chemotherapy with oxaliplatin-based combination therapy; Adjuvant chemotherapy with another protocol	In stage II adjuvant chemotherapy should be taken into consideration in selected risk situations (T4, tumour perforation/tears, surgery under emergency conditions, number of examined lymph nodes too small). [8.6.]	B/3b
#4	Rectal cancer 10 cm from the anal verge, pretherapeutic staging cT3 N + M0 CRM- EMVI- after long-term neoadjuvant radiochemotherapy (ARO scheme 50.4 Gy with Capecitabine) and postoperative UICC Stage I (ypT2 N0 (0/25) L0 V0 Pn0 R0 CRM-, Regression grade II), ECOG 0, Age 68	No adjuvant chemotherapy; Adjuvant chemotherapy with fluoropyrimidine monotherapy (oral/IV); Adjuvant chemotherapy with oxaliplatin-based combination therapy; Adjuvant chemotherapy with another protocol	A recommendation for or against adjuvant chemotherapy following neoadjuvant radiochemotherapy cannot be given on the basis of the available data for rectal cancer. [8.33.]	0/5
#5	Rectal cancer 10 cm from the anal verge, pretherapeutic staging cT3 N0 M0 CRM- EMVI- after short-term radiotherapy (5 × 5 Gy) and postoperative UICC Stage III (ypT3 N1 (2/25) L0 V0 Pn0 R0, CRM- EMVI-), ECOG 0, Age 68	No adjuvant chemotherapy; Adjuvant chemotherapy with fluoropyrimidine monotherapy (oral/IV); Adjuvant chemotherapy with oxaliplatin-based combination therapy; Adjuvant chemotherapy with another protocol	A recommendation for or against adjuvant chemotherapy following neoadjuvant radiochemotherapy cannot be given on the basis of the available data for rectal cancer. [8.33.]	0/5
**Visceral oncological case vignettes on adjuvant chemotherapy (evidence-based recommendations)**				
#6	Rectal cancer 10 cm from the anal verge, pretherapeutic staging cT1 N0 M0 CRM- EMVI- and postoperative Stage I (pT1, N0 L0 V0 R0, G2), ECOG 0, Age 68 [No adjuvant chemotherapy; Adjuvant chemotherapy with infusional 5-FU; Adjuvant chemotherapy with capecitabine (mono); Adjuvant chemotherapy with XELOX; Adjuvant chemotherapy according to FOLFOX]	No adjuvant chemotherapy; Adjuvant chemotherapy with infusional 5-FU; Adjuvant chemotherapy with capecitabine (mono); Adjuvant chemotherapy with XELOX; Adjuvant chemotherapy according to FOLFOX	In UICC stage I (pT1/2N0), R0 resection may not be followed by adjuvant therapy. [8.30.]	A/1b
#7	Colon cancer of the left flexure, postoperative Stage III (pT3, N2 (4/28) L0 V1 R0, G3), ECOG 0, Age 60 [No adjuvant chemotherapy; Adjuvant chemotherapy with infusional 5-FU; Adjuvant chemotherapy with capecitabine (mono); Adjuvant chemotherapy with XELOX; Adjuvant chemotherapy according to FOLFOX]	No adjuvant chemotherapy; Adjuvant chemotherapy with infusional 5-FU; Adjuvant chemotherapy with capecitabine (mono); Adjuvant chemotherapy with XELOX; Adjuvant chemotherapy according to FOLFOX	For adjuvant chemotherapy of stage III colon cancer, a therapy containing oxaliplatin shall be given. [8.9.]	A/1b
Visceral surgical case vignettes on the localization-dependent choice of surgical approach				
#8	Preferred resection technique for a patient (BMI 25, no previous surgery) with a colorectal carcinoma cT3 N1 in the following localization: Colon ascendens (#8.1), Flexura coli dextra (#8.2), Colon transversum (#8.3), Flexura coli sinistra (#8.4), Colon descendens (#8.5), Colon sigmoideum (#8.6), Rectum 14 cm from the anocutaneous line (#8.7), Rectum 8 cm from the anocutaneous line (#8.8), Rectum 3 cm from the the anocutaneous line (#8.9)	Laparoscopic; Robotic; NOTES; Open	Laparoscopic colon and rectal cancer resections can be performed with comparable results to open surgical techniques if the surgeon has appropriate expertise and the selection is appropriate. [7.46.]; Recent operation procedures (e.g. robotics, NOTES) cannot be recommended, because of insufficient data outside of studies. [7.48]	A/1a; B/NA (only expert consensus)

After the literature research of the guideline, the study by Grothey et al. (2018) was published, which added evidence to the treatment decision for adjuvant chemotherapy in stage III colon cancer patients (#7). The case descriptions follow the TNM Classification of Malignant published by the Union for International Cancer Control Tumors

^a^The case vignettes are translated from the German questionnaire. The guideline recommendations are taken from the English version of the Evidenced-based Guideline for Colorectal Cancer of the German Guideline Program in Oncology (GGPO) with the AWMF-Registration Number: 021/007OL

^b^To classify the distortion risk of the identified studies, the guideline uses the system of the Oxford Centre for Evidence-based Medicine version 2009 (available under www.cebm.net). Recommendations are classified as expert consensus if no literature research was performed. In this cases the methodology of the grade of recommendation is based on the AWMF-rules, which require a formal consensus process. The result of each vote (degree of consensus) is categorized in the Grades A (Strong recommendation), B (Recommendation) and 0 (Recommendation open).

### Survey distribution and data collection

The survey was conducted as a standardized online survey using the web application and cloud service SoSci Survey, which is a widely used German survey tool designed for academic and scientific research [27]. We contacted the coordinators of all 314 certified colorectal cancer centers in Germany (*n* = 296), Austria (*n* = 3), and Switzerland (*n* = 15) with an email containing the URL of the study website and asked them to participate in the survey with their tumor boards. If the vignettes could not be discussed in a tumor board meeting the coordinators could alternatively answer the survey themselves reflecting the most likely decision of their tumor board, or delegate the survey (or parts of it) to a deputy with comparable expertise. In that case, the person filling out the survey was encouraged to choose the answers as the tumor board would typically do. Next to the study information, the website offered two options: 1. to fill out the survey online or 2. to download and print out a PDF file of the survey. If the second option was selected, the filled-out document could then be uploaded to the website again. This was implemented to facilitate the answering within the tumor board meeting. The PDF file of the questionnaire in German is attached in Supplement 1. For online participants, the start time of the survey, the time of the last entry and the time spent on each survey page were recorded. Apart from this, no metadata was saved and no browser cookies were set. The website could be accessed multiple times through an open link. Therefore, the consecutive responses were checked for duplicates. The final data set was also tested for duplicate rows.

All participants were reminded two times (after 2 and after 8 weeks). In addition, the Working Group of DKG-certified German Colorectal Cancer Centers, which represents a large number of the centers, announced the survey (4 days before the start of the survey) and sent an additional non-personalized reminder to its members after 5 weeks. The survey period was November 14, 2023, through January 31, 2024.

The data were collected anonymously so that the answers could not be linked to the respondents or their institution. The hosting study onkoFDZ was funded by the German Ministry of Health. The study protocol was reviewed by the Ethics Committee of the University of Regensburg and approved without the need for a vote in accordance with Article 15 of the Professional Code for Physicians in Germany.

### Statistical analyses

The survey questions regarding the visceral oncological and the visceral surgical case vignettes were analyzed separately in a complete case analysis. The centers had to complete at least one of the two parts of the survey to be included in the analysis. The data were analyzed using descriptive statistics. For each vignette, heterogeneity was quantified using the Miller-Madow entropy estimator [28] with 95% bootstrap confidence intervals (10,000 replications) [29]. Normalized entropy estimates quantify the degree of uncertainty or disagreement in categorical data on a standardized 0 to 1 scale [18]. A normalized entropy (NE) of 0 indicated the maximum agreement of responses and a NE of 1 represents an equal distribution across all possible responses. For example, a NE of 0.40 might correspond to a scenario in which a clear majority of respondents (e.g., 70%) prefer one treatment option, while the remaining preferences are distributed among alternative options (e.g., 20% and 10%). This pattern suggests moderate consensus within the group. In contrast, an NE of 0.70 could arise when preferences are more evenly dispersed, such as 40% selecting one option, 30% another, 20% a third, and 10% a fourth, reflecting substantial heterogeneity and the absence of a dominant preference. Entropy measures were selected over variance-based metrics because they directly capture uncertainty in categorical preference distributions, making them more interpretable for multinomial responses. The Miller-Madow estimator was used due to the incorporated bias correction term for entropy estimation in smaller samples. Ties in categorical responses were inherently addressed through frequency aggregation using observed category counts, with explicit inclusion of zero-count categories. The differences between various institutional groups of centers (e.g. university hospitals) were analyzed exploratively to generate further hypothesis. For reporting the possible combinations of localization-dependent surgical approaches we used UpSet plots [30].

All analyses were performed using R 4.4.1 [31] and primarily the software packages entropy 1.3.1 by Hausser & Korbinian [32] for the NE calculations as well as tidyverse 2.0.0 by Wickham et al. [33] and gtsummary 1.7.2 by Sjoberg et al. [34] for preparing, reporting and visualizing the results.

## Results

### Response and hospital characteristics

**Fig. 1:**
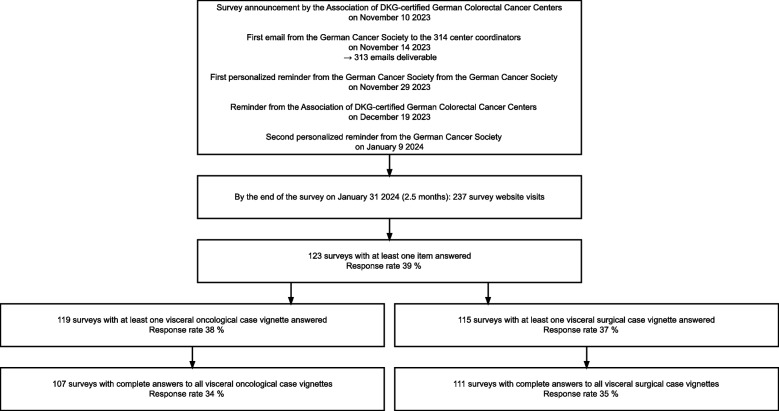
Data flowchart of the survey process and sample selection Note. The online survey was conducted using the web application and cloud service SoSci Survey (www.soscisurvey.de). The website could be accessed multiple times through an open link

A total of 123 centers responded to the survey (response rate 39%). Of these, 107 centers completed the visceral oncological case vignettes and 111 completed the visceral surgical case vignettes (Fig. 1). The majority of responses (64%) came from doctors representing a tumor board. Half of the centers were oncological centers, meaning they were not exclusively certified for the treatment of colorectal cancer, but rather bundle the diagnosis and treatment of several oncology specialties. In total, 183 (58%) of the colorectal cancer centers in Germany were also oncology centers, 8% more than in the responding sample. Furthermore, the vast majority were teaching hospitals (81%), 12% were university hospitals and 6% were Comprehensive Cancer Centers designated by the German Cancer Aid [25], (Table 2). At the time of the survey, there were 38 university hospitals (12% of all colorectal cancer centers) and 14 CCCs in Germany. The CCCs comprised 25 individual certified colorectal cancer centers, accounting for 8% of all colorectal cancer centers. The centers reported a median number of 80 (IQR: 65–100, Range: 45–200) patients with primary colorectal cancer treated in 2022. Supplement 2 shows a comparison of the number of patient cases between the study sample and all certified colorectal cancer centers. The mean case number of the study sample was 10 cases higher than the mean of primary cases across all centers.

**Table 2:** Characteristics of the sample

Center characteristics	*N* = 115
Fully answered case vignettes, n (%)	
Response to all case vignettes	103 (89.6)
Responses to visceral oncological case vignettes exclusively	4 (3.5)
Responses to visceral surgical case vignettes exclusively	8 (7.0)
Tumor board or deputy response, n (%)	
Actual response of a tumor board	41 (35.7)
Response of the center coordinator or deputy on behalf of the tumor board	74 (64.3)
Center type (Multiple answers possible)	
University hospital, n (%)^a^	14 (12.4)
Teaching hospital, n (%)	92 (81.4)
Oncological center^b^, n (%)	57 (50.4)
Comprehensive Cancer Center (funded by German Cancer Aid)^c^, n (%)	7 (6.2)
Missing	2
Number of primary operative colorectal cancer cases, n (%)	
> 100 cases	27 (25.0)
75 to 100 cases	31 (28.7)
< 75 cases	50 (46.3)
Missing	7

Centers reported a median number of 80 (IQR: 65–100, Range: 45–200) patient cases with primary colorectal cancer treated in 2022. Supplement 2 shows a comparison of the number of patient cases between the study sample and all certified colorectal cancer centers

^a^The total number of certified colorectal cancer centers at university hospitals in Germany at the time of the survey was 38 (12.1% of all certified colorectal cancer centers)

^b^All centers in this study are cancer centers certified by the German Cancer Society and thereby are specialize in the treatment of colorectal cancer. The status"oncological center"means that a center is certified for the treatment of several tumor entities. A visceral oncology center consists of at least one colorectal cancer center, that has an additional certificate for treating one or more of the tumor entities: liver, stomach, pancreas, oesophagus or anal carcinoma. The total number of oncology centers for visceral surgery in Germany at the time of the survey was 183 (58.3%)

^c^The Comprehensive Cancer Centers (CCC), funded by the German Cancer Aid are affiliated with universities and have a special focus on research and cross-regional oncological care. The total number of CCCs in Germany at the time of the survey was 14, with 25 individual certified colorectal cancer centers (8.0% of all certified colorectal cancer centers)

### Visceral oncological case vignettes

**Fig. 2:**
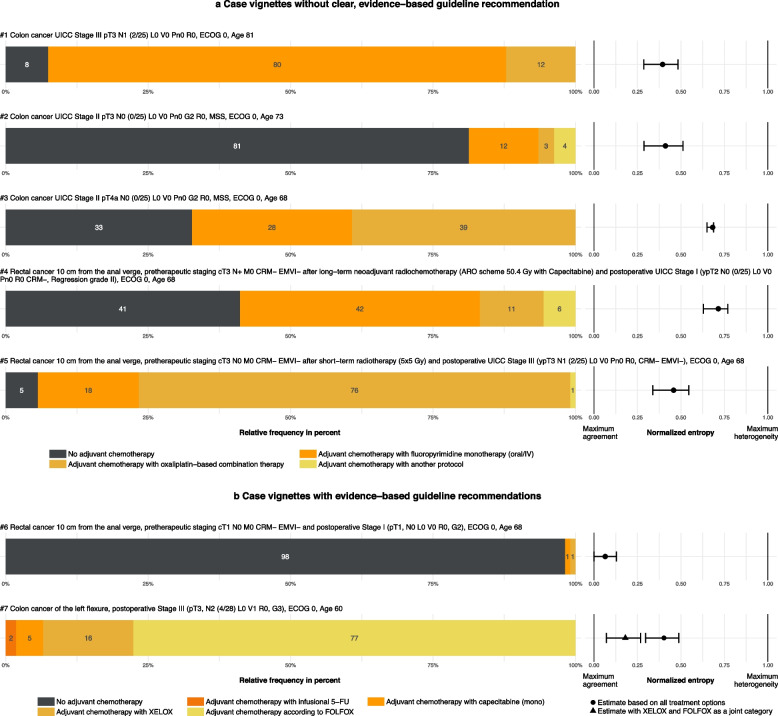
Treatment preferences of the visceral oncological tumor boards and their deputies Note. Frequency values in cases of unclear (**a**) and evidence based (**b**) guideline recommendation are reported within the bars in percent. Entropy estimates show the heterogeneity between 0 (Maximum agreement of responses) and 1 (Equal distribution across all 4 (#1–5) or 5 (#6–7) possible responses). In order to illustrate the heterogeneity with regard to the guideline recommendation 8.9., an additional estimate was given for vignette #7 in which the oxaliplatin-containing treatment options (XELOX and FLOFOX) were jointly categorized. Exact estimates and 95% confidence intervals are reported in Supplement 7A. *N* = 107, Response of tumor boards: *n* = 37, Responses of the center coordinators or deputies on behalf of the tumor board: *n* = 70

Figure 2a shows the 5 case vignettes for which there is limited or no conclusive evidence regarding the benefit of adjuvant chemotherapeutic treatment. Vignette #1 on the treatment of the elderly (age 81) with UICC stage III colon cancer shows a clear preference (80%) for adjuvant treatment with fluoropyrimidine monotherapy (oral or intravenous therapy) and a NE of 0.39 (95% CI 0.29–0.48). Seven percent would have refrained from any form of adjuvant chemotherapy. In vignette #2 on UICC II pT3 colon cancer patients, 81% favored no adjuvant treatment (NE 0.41; 95% CI 0.28–0.51). Nevertheless, a substantial proportion (19%) of the centers would perform adjuvant chemotherapy, although the recommendation grade 0 leaves the recommendation open. The centers that selected a different protocol reported either the use of CAPOX (capecitabine combined with oxaliplatin) or would have included the patients in the Circulate study, where they are tested for circulating tumor deoxyribonucleic acid after surgery, which serves as a selection criterion for chemotherapy. When a risk factor (pT4a) was present in a comparable cohort of colon cancer patients in vignette #3, the heterogeneity was higher (NE 0.68; 95% CI 0.63–0.77) with just one-third favoring no adjuvant chemotherapy, 28% preferring fluoropyrimidine monotherapy and 39% preferring oxaliplatin-based combination therapy. In the rectal cancer vignettes evaluating unclear guideline recommendations, the degree of heterogeneity in treatment preference varied comparably. Vignette #4 on rectal cancer patients with postoperative UICC Stage I after neoadjuvant treatment of initially lymph node-positive UICC Stage III showed high variation (NE 0.71; 95% CI 0.63–0.77) with 41% preferring no adjuvant chemotherapy, 42% preferring fluoropyrimidine monotherapy and 11% preferring oxaliplatin-based combination therapy as adjuvant treatment. Centers who chose a different adjuvant option in vignette #4 (6%) specified in the free text field that they favored a completion of the neoadjuvant treatment with capecitabine. In vignette #5 however, only 6% would not perform any form of adjuvant chemotherapy in patients having lymph-node positive postoperative UICC Stage III. Thus, this vignette showed a lower heterogeneity (NE 0.46; 95% CI 0.34–0.55) with 76% preferring an oxaliplatin-based combination therapy.

Figure 2b shows the 2 case vignettes used as control cases. Vignette #6 thereby shows an almost perfect agreement (NE 0.06; 95% CI 0.00–0.13) with 98% of all centers acting in accordance with the evidence-based guideline recommendation to refrain from adjuvant chemotherapy in patients with small pT1 lymph-node negative rectal carcinoma. A more differentiated pattern can be seen in vignette #7 (NE 0.40; 95% CI 0.29–0.49). Here 78% answered in accordance with the latest study by Grothey et al. (2018). This study added evidence to the treatment decision for adjuvant chemotherapy with oxaliplatin in stage III colon cancer patients after the literature research of the guideline was conducted. When the oxaliplatin-containing treatment options recommended in the guideline were considered as a single category, there was substantially less heterogeneity (NE 0.21; 95% CI 0.08–0.31). The guideline-based recommendation of adjuvant chemotherapy with XELOX (capecitabin and oxaliplatin) was followed by 16% of the centers. A proportion of 7% of the centers chose answers that were neither in line with the (outdated) guideline recommendation nor with the current evidence.

In the exploratory analyses (Supplement 3) of the distributions of preferred treatment options among the dichotomous groups:"Actual response of a tumor board or of a center coordinator/deputy","University hospital status","Teaching hospital status","Oncological center","Comprehensive Cancer Center (funded by German Cancer Aid)” and"Caseload (≥ 85 vs < 85)"no consistent patterns were found. Supplement 4 A–D shows the results of the tumor board responses and the deputy responses individually for the vignettes #1–7. The exploratory analysis additionally displays the distribution of missing values from centers that responded to at least one visceral oncology case vignette.

### Visceral surgical vignettes

**Fig. 3:**
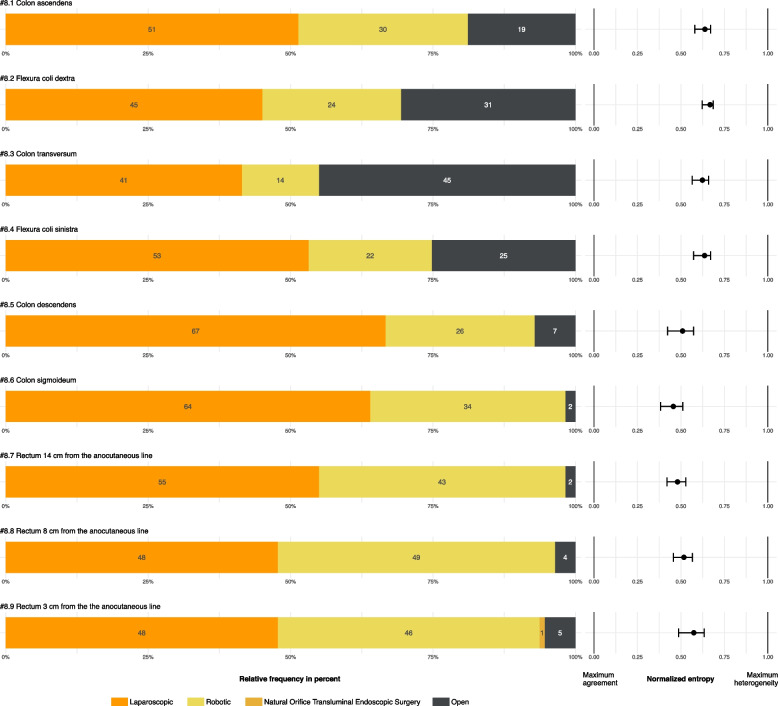
Preferred resection technique of the visceral surgery department and their deputies Note. Frequency values of preferred resection technique for a patient (BMI 25, no previous surgery) with colorectal cancer cT3 N1 depending on the localization are reported within the bars in percent. Entropy estimates show the heterogeneity between 0 (Maximum agreement of responses) and 1 (Equal distribution across all 4 possible responses). Exact estimates and 95% confidence intervals are reported in Supplement 7B. *N* = 111, Response of tumor boards: *n* = 40, Responses of the center coordinators or deputies on behalf of the tumor board: *n* = 71

The visceral surgical vignettes (Fig. 3) addressed the localization-dependent choice of surgical access, with a preference for laparoscopic and robotic approaches. An exception was the surgery of the colon transversum (#8.3): here a smaller proportion (14% less than in every other localization) of visceral surgery departments would perform a minimally invasive procedure. The NE ranged from 0.46 (95% CI 0.38–0.51) to 0.67 (95% CI 0.62–0.69) with higher estimates for the vignettes #8.1–8.4.

All but one of the surgical departments (*n* = 14) that chose"robotic"as the answer for the colon transversum also did so for every other localization (Supplement 5). The same pattern is evident for laparoscopic procedures, where only one department that chose"laparoscopic"for the colon transversum would perform open surgery for another localization. The remaining departments that chose"laparoscopic"(*n* = 45) preferred minimally invasive techniques for every other tumor localization surveyed. Of the 30 centers reporting the intention to change their surgical technique within the next 12 months, 24 (80%, multiple answers possible) wanted to perform more robotic procedures and 13 (43%, multiple answers possible) wanted to perform more laparoscopic procedures. NOTES was only selected once as an option for resection in the distal third of the rectum.

Similar to the results of the oncological vignettes no pattern of systematic differences between institutional groups was found in the use of minimal invasive surgery techniques (Supplement 6). Information on the distribution of missing values from centers that responded to at least one visceral surgical case vignette is also included in Supplement 6. Supplement 4 E–H shows the results of the tumor board responses and the deputy responses individually for the vignettes #8.1–8.9.

## Discussion

This case vignette study provides a comprehensive description of the distribution of institutional treatment preferences for colorectal cancer in the absence of clear evidence-based recommendations. It refers to DKG-certified colorectal cancer treatment as a use case since the German guideline gives no clear advice for certain aspects, such as adjuvant chemotherapy in elderly patients or concerning the most appropriate surgical approach [3]. The present study shows that there is considerable heterogeneity in such situations, as different institutions prefer different treatment options for the same—hypothetical—patient. This is not a sign of poor treatment quality but rather has to be attributed to the fact that the lack of guideline recommendations is a consequence of currently insufficient scientific evidence. When randomized trials are increasingly difficult to realize or even inappropriate due to the nature of the topic of interest [16], knowledge about institution-specific treatment preferences can be used to inform scientific projects analyzing clinical routine data.

It is widely consented that clinical decision-making should rely on scientific evidence. However, there are innumerable situations in which either available studies contradict each other or in which there are no (reliable) studies at all on a particular topic. Notwithstanding this problem, treatment decisions must be made. These decisions are then inevitably based on other resources such as existing experiences, beliefs and individual perceptions [13, 14] often shaped by the institutional level [6, 7]. The process of deciding on a suitable therapy may be driven more by factors such as personality and behavioral traits based on experience with specific treatments or patient populations [13, 35]. For instance, clinicians who are more experienced in treating a particular patient population or who are more willing to take risks may be more likely to favor the use of a therapy, while less experienced or risk-averse clinicians may not share this preference. At the level of multidisciplinary tumor boards, cultural factors such as the allocation of tasks, the general communication and leadership style within the institution or the assertiveness of a particular discipline or expert might influence the preference [36]. At a broader institutional level, certain differences in training backgrounds, subspecialty expertise, or continuing education could influence treatment preferences [19]. This process may be reinforced by norms of efficiency that lead to similar treatment patterns being carried out repeatedly. Also, insufficient evidence may be more easily interpreted differently at a personal or organizational level. As part of the shared decision-making process, a final treatment decision is then made together with the patient based on the tumor board's recommendation [37]. Consequently, patients will have different chances to receive a certain kind of therapy depending on the hospital in which they undergo their treatment. The majority of patients will likely be unaware of this fact, as most treatment decisions in oncology, e.g., the choice of a chemotherapy substance, require highly specialized knowledge that cannot be judged by a layperson. Even though the institutions surveyed were explicitly asked to state their own treatment preference, the results raise important ethical questions regarding patient autonomy. The differences in treatment preference could lead to different advice for patients, which has a substantial influence on the patient's decision. Importantly, institutional treatment preference must be considered a latent variable that remains implicit until the patient or another person involved in the treatment decision process asks for explication. It also remains implicit whether the assumed institutional preference differs from the preference of an individual doctor or patient and may be overruled. Thus, it can be expected that patients choose their hospital on the basis of other factors, such as its proximity to their place of residence, rather than certain treatment preferences [38]. Adapting the patients’ perspective, is a perfectly sensible approach, since evidence is missing as to whether one of the available therapy options is superior over the others.

However, the heterogeneity of treatment preference on the institutional level might be a crucial factor contributing to improving the quality of routine-data-based analyses, which in many cases might represent the best achievable evidence [39]. Since therapy allocation cannot be randomized in retrospective, observational studies have often been criticized for being prone to confounding-by-indication and similar problems [40]. Often it is not possible to account for all factors influencing therapy decisions due to unavailability. This issue is usually referred to as “unobserved confounding”. The results of the present study show that patient- and disease-related factors are not the only factors influencing the decision on which therapy is administered. The fact that different institutions prefer different therapy options for patients with identical characteristics may therefore be a valuable piece of information for retrospective routine data analyses. Institution-specific treatment preferences can serve as a basis to address confounding-by-indication in observational data, when they are associated with the treatment, are independent from the outcome and do not affect it other than through the treatment [41, 42]. Machine learning approaches may help to process and synthesize routine data containing all kinds of relevant information on patients, their disease, comorbidities, previous treatments and medical history, and the institutions they were treated in [43]. Thereby, it might be possible to reconstruct an institution’s treatment preference as a latent variable in specific situations. Deviance or concordance on patient level may consequently serve as an instrument variable and can be included in the statistical analyses. Such information could contribute to enhance the quality of observational analyses considerably.

Reconstructing and using information about treatment preferences (in the sense of both intended and definite treatment) is one of the central concepts of the onkoFDZ project [15]. An indispensable prerequisite to use institutional-level treatment preferences is the existence of heterogeneity [20]. In order to estimate treatment effects in this way, the three main assumptions of the instrumental variable must further be met. First, the instrument variable must be associated with the treatment (relevance). Second, it must not influence the outcome other than through the treatment (exclusion restriction). And third, it must have no common cause with the outcome (independence) [20, 44]. With regard to the independence assumption, it is important to distinguish between the preferences surveyed in this study and the tumour board preferences captured in routine data, which would typically be used as an instrumental variable. The institution’s treatment preference, as reconstructed from routine data, is determined by the underlying preference of the tumour board, as well as by the clinical characteristics and preferences of the patients. Therefore, a further comparison with routine data is necessary to fully test the assumptions and to address possible confounding by case-mix due to differing patient collectives of the centers [41, 44]. The results presented in this paper have a high internal validity, which is indicated by the high homogeneity concerning the preferred therapy options in the control cases. In the first control vignette (#6) 98% answered according to the guideline recommendation. In the second control vignette (#7), 94% chose an oxaliplatin-containing therapy as recommended in the guideline, with 78% choosing FOLFOX over XELOX, as suggested in a study published after the last guideline update [26]. Concerning the surgical vignettes, there exists great consistency of the preferred approach over the different tumor locations, which is also a sign of high reliability. However, differences in preferences should not be generalized to differences in clinical practice due to the use of case vignettes to capture tumor board preferences. While this is a well-established methodology for assessing clinical judgment in a controlled setting, the simplified and standardized nature of vignettes cannot capture the full spectrum of factors that influence treatment decisions in routine clinical practice, which leads to an over- or underestimation of heterogeneity in treatment prescription. Actual clinical decisions may therefore differ from, but are influenced by, the preference obtained from the simplified vignettes. It should also be noted that although there was no difference in the preference for adjuvant chemotherapy or minimally invasive surgery between tumor boards and individuals representing a tumor board (Supplement 3 A, Supplement 6 A), only 36% of centers discussed the case vignettes within a tumor board meeting. This can potentially introduce bias into the results, as individual coordinators may not accurately represent the collective decision-making process of a multidisciplinary tumor board. In a previous study of the same target population, in which also the center coordinators were contacted, mainly senior doctors of the surgical department (85%) and the internal medicine department (10%) responded to the survey [45]. This survey did not collect information on deputies'qualifications or roles. The preferences shown are presumably time-dependent. Vignette #7 indicates this, as more recent evidence leads to a more differentiated preference than in Vignette #6. Longitudinal study designs should assess the temporal stability of institutional treatment preferences and identify the factors driving changes to these preferences over time. As the distribution of preferred treatments may change over time, while heterogeneity, and thus the utility of treatment preferences as an instrument variable, may remain unchanged, further studies should report comparable entropy estimates for reported preferences. Another considerable strength of the present study is the large number of addressed institutions and the response rate. All colorectal cancer centers in Germany which account for approximately 50% of colorectal cancer treatment cases [46] and some other DKG-certified colorectal cancer centers from neighboring countries received the questionnaire. 39% of the centers reacted to the questionnaire, which is a remarkably high response rate for questionnaire-based studies. Nevertheless, there is considerable potential for selection bias, as participating centers may differ systematically from non-participating centers. For instance, more research-active centers may be more likely to participate in the study than centers with fewer resources or involvement in research activities. The higher average volume of 10 cases per year among participating hospitals, compared to all certified colorectal cancer centers, could be an indicator of that. The other hospital characteristics of the sample (University, Comprehensive Cancer Center and Oncological center status) showed good comparability to the population in terms of frequency, which indicates good representativeness. The remaining differences could influence the reported treatment preferences and both over- and underestimate heterogeneity. Even though the survey was anonymous and there was an option not to answer individual questions, there may have been a response bias, as the participants knew that the survey was intended to reflect their institution-specific treatment preferences. Therefore, there may have been a tendency to choose particularly innovative treatment measures or to be as consistent as possible in the response options. Additionally, a recency bias could have affected the validity of vignette responses as representation of the institutions typical decision-making pattern. The possible influence of tumor board members’ treatment preferences by recent patient outcomes, adverse events, or particularly memorable cases was not accounted for in this study. It was also not possible to differentiate the composition of the preferences shown at individual, team and broader institutional level. To expand on the results, it would have been interesting to include other, non-certified hospitals in this study, but there exists no comprehensive list of all hospitals providing colorectal cancer treatment. Subgroup analyses of institutional factors could also provide further information on the generalizability of the results, which were only performed exploratively in this study. A subsequent study could try to identify at least all hospitals involved in colorectal cancer treatment in a selected region, e.g. through the German Hospital Federation or a cancer registry. In addition, the generalizability of the results to other contexts and healthcare systems with resource constraints, such as access to chemotherapeutic agents or robotic surgical systems, should be examined. Future studies might also aim to find out the institution-specific rationale behind the treatment preference, which was deliberately omitted in this study to guarantee the participants’ anonymity and spare them efforts. Qualitative study designs could help explain the reasons behind certain treatment preferences and identify the underlying beliefs, attitudes and contextual factors that influence the decision-making process. In order to better understand the institutional component of the preference differences, the differentiation between individual and institutional treatment preference should be examined in more detail. Finally, further research should attempt to examine the influence of treatment preference on patient outcomes by linking practitioner survey data to treatment data and patient reported outcomes in order to better understand whether heterogeneity in treatment preference also leads to differences in outcomes.

## Conclusion

In prototypical case vignettes without clear, evidence-based guideline recommendations, pronounced heterogeneity of treatment preferences between centers was found. Institution-specific treatment preferences play an important role in clinical decision-making in the absence of clear guideline recommendations. Reconstructing and using information about these general treatment preferences together with the definite treatment as reported in routine data can contribute to enhance the quality of evidence derived from observational studies considerably. This seems especially important in the context of clinical questions for which no randomized trials are possible and analyses based on clinical routine data represent the best available source of evidence.

## Supplementary Information

Supplementary Material 1

Supplementary Material 2

Supplementary Material 3

Supplementary Material 4

Supplementary Material 5

Supplementary Material 6

Supplementary Material 7

## Data Availability

The dataset generated and analyzed during the current study is not publicly available in order to protect the privacy of the surveyed institutions.
