# Supplementary material for: Heterogeneity of treatment preferences in the absence of guideline recommendations – a case vignette study in colorectal cancer tumor boards in Germany, Austria and Switzerland
Source: BMC Gastroenterol. 2025 Oct 7;25:700. doi: 10.1186/s12876-025-04183-5 (PMC12505869; doi:10.1186/s12876-025-04183-5)
Supplement: Supplementary file 1 — Supplementary Material 1 [file 12876_2025_4183_MOESM1_ESM.pdf]

Befragung institutionsspezifische Behandlungspräferenz

Vielen Dank für Ihre Teilnahme!

Bitte füllen Sie den Fragebogen möglichst vollständig aus und senden Sie uns Ihre Antworten über **einen der 3 Wege anonym** zurück.

1. **Antworten am PC in den digitalen Fragebogen übertragen**

ODER

2. **Ausgefüllten Fragebogen einscannen und PDF Datei auf der 2. Seite der Befragung (*Fragebogenformat* → *Schriftlich*) hochladen**

ODER

3. **QR-Code scannen und Antworten in den digitalen Fragebogen übertragen**

Der Zeitaufwand, die Ergebnisse zu übertragen, beträgt etwa 3 Minuten.

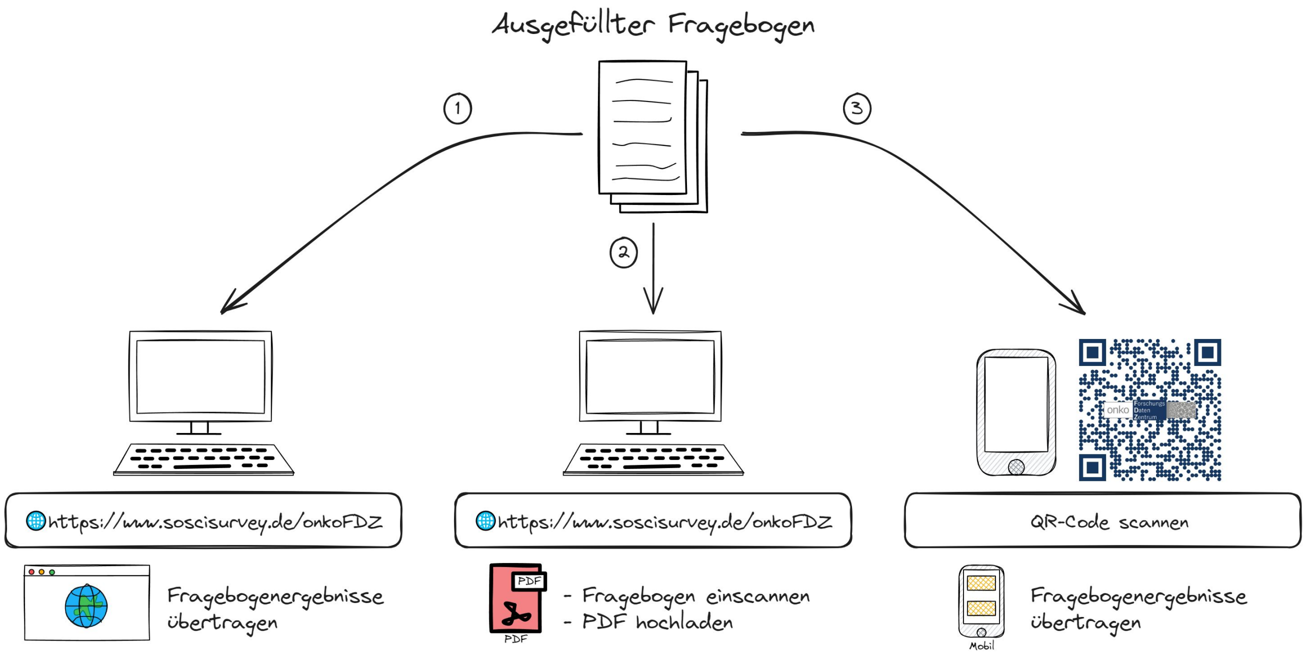

Viszeralonkologie

Bitte wählen Sie die Antwort aus, die am ehesten einer **typischen Empfehlung Ihres Tumorboards** entspricht und mit der Sie gemeinsam mit Ihrer Patientin oder Ihrem Patienten in die partizipative Entscheidungsfindung einsteigen würden.

1. Würden Sie bei einem 81-jährigen Patienten (m/w), ECOG 0, mit Kolonkarzinom UICC Stadium III pT3 N1 (2/25) L0 V0 Pn0 R0 in der Regel eine adjuvante Chemotherapie durchführen?

- ◯ Nein
- ◯ Ja, mit Fluoropyrimidin-Monotherapie (oral/i.v.)
- ◯ Ja, mit Oxaliplatin-basierter Kombinationstherapie
- ◯ Ja, aber systemische Therapie mit anderem Protokoll
  optional: Angabe des Protokolls:

2. Würden Sie bei einem 73-jährigen Patienten (m/w), ECOG 0, mit Kolonkarzinom UICC Stadium II pT3 N0 (0/25) L0 V0 Pn0 G2 R0, MSS in der Regel eine adjuvante Chemotherapie durchführen?

- ◯ Nein
- ◯ Ja, mit Fluoropyrimidin-Monotherapie (oral/i.v.)
- ◯ Ja, mit Oxaliplatin-basierter Kombinationstherapie
- ◯ Ja, aber systemische Therapie mit anderem Protokoll
  optional: Angabe des Protokolls:

3. Würden Sie bei einem 68-jährigen Patienten (m/w), ECOG 0, mit Kolonkarzinom UICC Stadium II pT4a N0 (0/25) L0 V0 Pn0 G2 R0, MSS in der Regel eine adjuvante Chemotherapie durchführen?

- ◯ Nein
- ◯ Ja, mit Fluoropyrimidin-Monotherapie (oral/i.v.)
- ◯ Ja, mit Oxaliplatin-basierter Kombinationstherapie
- ◯ Ja, aber systemische Therapie mit anderem Protokoll
  optional: Angabe des Protokolls:

4. Würden Sie bei einem 68-jährigen Patienten (m/w), ECOG 0, mit Rektumkarzinom 10 cm ab ano, prätherapeutisches Staging cT3 N+ M0 CRM- EMVI- nach neoadjuvanter Langzeit-Radiochemotherapie (ARO-Schema 50,4 Gy mit Capecitabin) und postoperativem UICC Stadium I (ypT2 N0 (0/25) L0 V0 Pn0 R0 CRM-, Regressionsgrad II) in der Regel eine adjuvante Chemotherapie durchführen?

- ○ Nein
- ○ Ja, mit Fluoropyrimidin-Monotherapie (oral/i.v.)
- ○ Ja, mit Oxaliplatin-basierter Kombinationstherapie
- ○ Ja, aber systemische Therapie mit anderem Protokoll
  optional: Angabe des Protokolls:

5. Würden Sie bei einem 68-jährigen Patienten (m/w), ECOG 0, mit Rektumkarzinom 10 cm ab ano, prätherapeutisches Staging cT3 N0 M0 CRM- EMVI- nach neoadjuvanter Kurzzeit-Strahlentherapie - (5x5 Gy) und postoperativem UICC Stadium III (ypT3 N1 (2/25) L0 V0 Pn0 R0, CRM- EMVI-) in der Regel eine adjuvante Chemotherapie durchführen?

- ○ Nein
- ○ Ja, mit Fluoropyrimidin-Monotherapie (oral/i.v.)
- ○ Ja, mit Oxaliplatin-basierter Kombinationstherapie
- ○ Ja, aber systemische Therapie mit anderem Protokoll
  optional: Angabe des Protokolls:

6. Wie würden Sie bei einem 68-jährigen Patienten (m/w), ECOG 0, mit Rektumkarzinom 10 cm ab ano, prätherapeutisches Staging cT1 N0 M0 CRM- EMVI- und postoperativem Stadium I (pT1, N0 L0 V0 R0, G2) eine adjuvante Chemotherapie durchführen?

- ○ Nein
- ○ Ja, mit infusionalem 5-FU
- ○ Ja, mit Capecitabin (mono)
- ○ Ja, entsprechend XELOX
- ○ Ja, entsprechend FOLFOX

7. Würden Sie bei einem 60-jährigen Patienten (m/w), ECOG 0, mit Kolonkarzinom der linken Flexur und postoperativem Stadium III (pT3, N2 (4/28) L0 V1 R0, G3) eine adjuvante Chemotherapie durchführen?

- ○ Nein
- ○ Ja, mit infusionalem 5-FU
- ○ Ja, mit Capecitabin (mono)
- ○ Ja, entsprechend XELOX
- ○ Ja, entsprechend FOLFOX

Viszeralchirurgie

Die aktuelle S3-Leitlinie zum kolorektalen Karzinom gibt keine eindeutigen Empfehlungen zur Wahl des Operationszugangs bzw. der Operationstechnik.

Bitte wählen Sie im Folgenden diejenige Option aus, die am ehesten der für Sie **typischen Wahl des Operationszugangs Ihrer viszeralchirurgischen Abteilung** entspricht und mit der Sie in die partizipative Entscheidungsfindung gemeinsam mit Ihrer Patientin oder Ihrem Patienten gehen würden.

8. Wie würden Sie im Regelfall einen Patienten (m/w) mit einem kolorektalen Karzinom cT3 N1 und BMI 25, keine Vor-OPs in folgenden Lokalisationen operieren:

(Bitte eine Option ankreuzen)

C. ascendens	Laparoskopisch	Robotisch	NOTES	Offen
Re. Flexur	Laparoskopisch	Robotisch	NOTES	Offen
C. Transversum	Laparoskopisch	Robotisch	NOTES	Offen
Li. Flexur	Laparoskopisch	Robotisch	NOTES	Offen
C. descendens	Laparoskopisch	Robotisch	NOTES	Offen
Sigma	Laparoskopisch	Robotisch	NOTES	Offen
Rektum 14 cm a.a.	Laparoskopisch	Robotisch	NOTES	Offen
Rektum 8 cm	Laparoskopisch	Robotisch	NOTES	Offen
Rektum 3 cm	Laparoskopisch	Robotisch	NOTES	Offen

9. Planen Sie Ihre Technik innerhalb der kommenden 12 Monate zu ändern?

- ○ Ja, in mindestens einer der vorher genannten Lokalisationen
- ○ Nein

10. In welcher Form planen Sie, Ihre Technik innerhalb der kommenden 12 Monate zu ändern?

Falls Sie Frage 9 mit „Nein“ beantwortet haben, überspringen Sie bitte diese Frage.

(Mehrfachantwort möglich)

- ☐ mehr offen
- ☐ mehr laparoskopisch
- ☐ mehr robotisch

Klinikangaben

Bitte geben Sie uns darüber hinaus Auskunft, an welcher Art Klinik Sie tätig sind bzw. sich das Tumorboard befindet.

(Antwort freiwillig, Mehrfachantwort möglich/erforderlich)

11. Bitte machen Sie eine Angabe zur Anzahl der operativen Primärfälle Darmkrebs (Kolon + Rektum) an Ihrem Zentrum (Schätzung für 2022).

Anzahl der operativen Primärfälle Darmkrebs:

____

12. Bitte machen Sie eine Angabe über den Lehrstatus der Klinik, an welchem Ihr Zentrum angesiedelt ist.

- ☐ Universitätsklinik
- ☐ Lehrkrankenhaus
- ☐ Kein Lehrkrankenhaus

13. Bitte machen Sie eine genauere Angabe zum Status Ihres Zentrums.

- ☐ Organkrebszentrum
- ☐ Onkologisches Zentrum
- ☐ Onkologisches Spitzenzentrum (DKH-gefördert)

Vielen Dank für Ihre Teilnahme und das Interesse an unserer Studie!

Wir möchten uns ganz herzlich für Ihre Mithilfe bedanken.

Bitte scannen Sie den ausgefüllten Fragebogen ein und laden Sie die PDF-Datei auf der Befragungswebseite (https://www.soscisurvey.de/onkoFDZ) hoch oder übertragen Sie die Ergebnisse in die Onlinebefragung.

Falls Sie die Studienergebnisse dieser Studie erhalten möchten, haben Sie die Möglichkeit beim Hochladen Ihrer Antworten eine E-Mail-Adresse anzugeben.

Johannes Soff, Arbeitsgemeinschaft deutscher Darmkrebszentren – 2023
