# Supplementary material for: Heterogeneity of treatment preferences in the absence of guideline recommendations – a case vignette study in colorectal cancer tumor boards in Germany, Austria and Switzerland
Source: BMC Gastroenterol. 2025 Oct 7;25:700. doi: 10.1186/s12876-025-04183-5 (PMC12505869; doi:10.1186/s12876-025-04183-5)
Supplement: Supplementary file 2 — Supplementary Material 2 [file 12876_2025_4183_MOESM2_ESM.docx]

**Supplement 2**


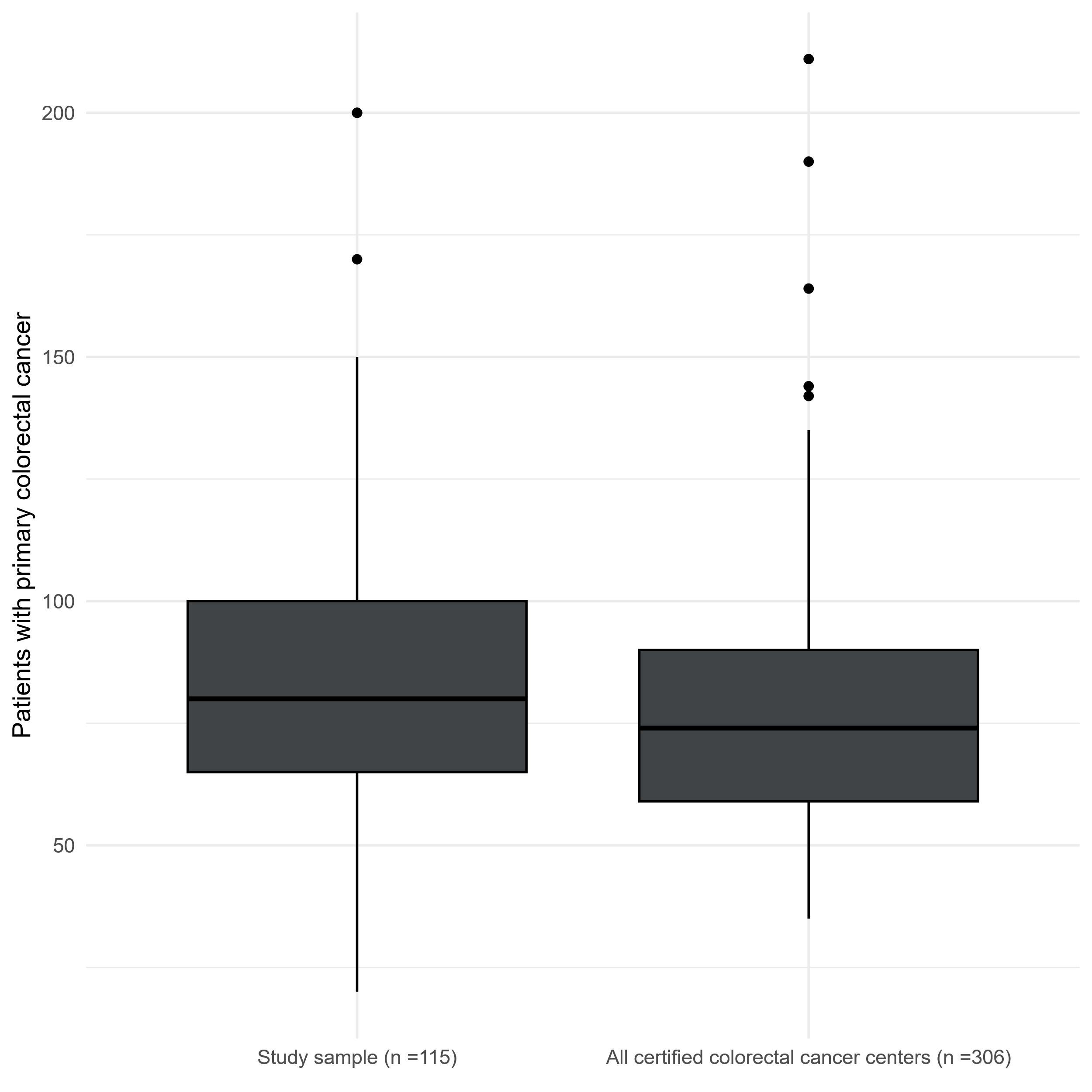


*Comparison of the number of patient cases with primary colorectal cancer treated in 2022 between the study sample and the entirety of certified colorectal cancer centers*

*Note.* Mean number of cases of all certified colorectal cancer centers: 76. Mean number of cases of the study sample: 86. One Sample t-test p-value = 0.003.
