# Supplementary material for: Heterogeneity of treatment preferences in the absence of guideline recommendations – a case vignette study in colorectal cancer tumor boards in Germany, Austria and Switzerland
Source: BMC Gastroenterol. 2025 Oct 7;25:700. doi: 10.1186/s12876-025-04183-5 (PMC12505869; doi:10.1186/s12876-025-04183-5)
Supplement: Supplementary file 3 — Supplementary Material 3 [file 12876_2025_4183_MOESM3_ESM.docx]

**Supplement 3**

**A**

| **Decision in favor of adjuvant chemotherapy in the case vignette,** **n (%)** | **Actual response of a tumor board**, N = 40 | **Response of the center coordinator or deputy on behalf of the tumor board**, N = 79 |
| --- | --- | --- |
| Colon cancer UICC Stage III pT3 N1 (2/25) L0 V0 Pn0 R0, ECOG 0, Age 81 | 35 (90%) | 73 (94%) |
| Missing | 1 | 1 |
| Colon cancer UICC Stage II pT3 N0 (0/25) L0 V0 Pn0 G2 R0, MSS, ECOG 0, Age 73 | 5 (13%) | 16 (21%) |
| Missing | 1 | 4 |
| Colon cancer UICC Stage II pT4a N0 (0/25) L0 V0 Pn0 G2 R0, MSS, ECOG 0, Age 68 | 25 (66%) | 50 (68%) |
| Missing | 2 | 6 |
| Rectal cancer 10 cm from the anal verge, pretherapeutic staging cT3 N+ M0 CRM- EMVI- after long-term neoadjuvant radiochemotherapy (ARO scheme 50.4 Gy with Capecitabine) and postoperative UICC Stage I (ypT2 N0 (0/25) L0 V0 Pn0 R0 CRM-, Regression grade II), ECOG 0, Age 68 | 23 (59%) | 44 (59%) |
| Missing | 1 | 5 |
| Rectal cancer 10 cm from the anal verge, pretherapeutic staging cT3 N0 M0 CRM- EMVI- after short-term radiotherapy (5x5 Gy) and postoperative UICC Stage III (ypT3 N1 (2/25) L0 V0 Pn0 R0, CRM- EMVI-), ECOG 0, Age 68 | 37 (95%) | 69 (95%) |
| Missing | 1 | 6 |
| Rectal cancer 10 cm from the anal verge, pretherapeutic staging cT1 N0 M0 CRM- EMVI- and postoperative Stage I (pT1, N0 L0 V0 R0, G2), ECOG 0, Age 68 | 1 (2.6%) | 1 (1.4%) |
| Missing | 1 | 5 |
| Colon cancer of the left flexure, postoperative Stage III (pT3, N2 (4/28) L0 V1 R0, G3), ECOG 0, Age 60 | 39 (100%) | 75 (100%) |
| Missing | 1 | 4 |

**B**

| **Decision in favor of adjuvant chemotherapy in the case vignette,** **n (%)** | **No University Hospital**, N = 101 | **University Hospital**, N = 14 |
| --- | --- | --- |
| Colon cancer UICC Stage III pT3 N1 (2/25) L0 V0 Pn0 R0, ECOG 0, Age 81 | 95 (94%) | 10 (77%) |
| Missing | 0 | 1 |
| Colon cancer UICC Stage II pT3 N0 (0/25) L0 V0 Pn0 G2 R0, MSS, ECOG 0, Age 73 | 18 (19%) | 2 (14%) |
| Missing | 5 | 0 |
| Colon cancer UICC Stage II pT4a N0 (0/25) L0 V0 Pn0 G2 R0, MSS, ECOG 0, Age 68 | 65 (69%) | 8 (57%) |
| Missing | 7 | 0 |
| Rectal cancer 10 cm from the anal verge, pretherapeutic staging cT3 N+ M0 CRM- EMVI- after long-term neoadjuvant radiochemotherapy (ARO scheme 50.4 Gy with Capecitabine) and postoperative UICC Stage I (ypT2 N0 (0/25) L0 V0 Pn0 R0 CRM-, Regression grade II), ECOG 0, Age 68 | 60 (62%) | 7 (50%) |
| Missing | 4 | 0 |
| Rectal cancer 10 cm from the anal verge, pretherapeutic staging cT3 N0 M0 CRM- EMVI- after short-term radiotherapy (5x5 Gy) and postoperative UICC Stage III (ypT3 N1 (2/25) L0 V0 Pn0 R0, CRM- EMVI-), ECOG 0, Age 68 | 90 (94%) | 14 (100%) |
| Missing | 5 | 0 |
| Rectal cancer 10 cm from the anal verge, pretherapeutic staging cT1 N0 M0 CRM- EMVI- and postoperative Stage I (pT1, N0 L0 V0 R0, G2), ECOG 0, Age 68 | 0 (0%) | 1 (7.1%) |
| Missing | 4 | 0 |
| Colon cancer of the left flexure, postoperative Stage III (pT3, N2 (4/28) L0 V1 R0, G3), ECOG 0, Age 60 | 98 (100%) | 14 (100%) |
| Missing | 3 | 0 |

**C**

| **Decision in favor of adjuvant chemotherapy in the case vignette,** **n (%)** | **No teaching hospital**, N = 23 | **Teaching hospital**, N = 92 |
| --- | --- | --- |
| Colon cancer UICC Stage III pT3 N1 (2/25) L0 V0 Pn0 R0, ECOG 0, Age 81 | 18 (82%) | 87 (95%) |
| Missing | 1 | 0 |
| Colon cancer UICC Stage II pT3 N0 (0/25) L0 V0 Pn0 G2 R0, MSS, ECOG 0, Age 73 | 3 (16%) | 17 (19%) |
| Missing | 4 | 1 |
| Colon cancer UICC Stage II pT4a N0 (0/25) L0 V0 Pn0 G2 R0, MSS, ECOG 0, Age 68 | 12 (63%) | 61 (69%) |
| Missing | 4 | 3 |
| Rectal cancer 10 cm from the anal verge, pretherapeutic staging cT3 N+ M0 CRM- EMVI- after long-term neoadjuvant radiochemotherapy (ARO scheme 50.4 Gy with Capecitabine) and postoperative UICC Stage I (ypT2 N0 (0/25) L0 V0 Pn0 R0 CRM-, Regression grade II), ECOG 0, Age 68 | 12 (63%) | 55 (60%) |
| Missing | 4 | 0 |
| Rectal cancer 10 cm from the anal verge, pretherapeutic staging cT3 N0 M0 CRM- EMVI- after short-term radiotherapy (5x5 Gy) and postoperative UICC Stage III (ypT3 N1 (2/25) L0 V0 Pn0 R0, CRM- EMVI-), ECOG 0, Age 68 | 19 (100%) | 85 (93%) |
| Missing | 4 | 1 |
| Rectal cancer 10 cm from the anal verge, pretherapeutic staging cT1 N0 M0 CRM- EMVI- and postoperative Stage I (pT1, N0 L0 V0 R0, G2), ECOG 0, Age 68 | 1 (5.3%) | 0 (0%) |
| Missing | 4 | 0 |
| Colon cancer of the left flexure, postoperative Stage III (pT3, N2 (4/28) L0 V1 R0, G3), ECOG 0, Age 60 | 20 (100%) | 92 (100%) |
| Missing | 3 | 0 |

**D**

| **Decision in favor of adjuvant chemotherapy in the case vignette,** **n (%)** | **No Comprehensive Cancer Center^a^,** N = 108 | **Comprehensive Cancer Center^a^**, N = 7 |
| --- | --- | --- |
| Colon cancer UICC Stage III pT3 N1 (2/25) L0 V0 Pn0 R0, ECOG 0, Age 81 | 99 (92%) | 6 (100%) |
| Missing | 0 | 1 |
| Colon cancer UICC Stage II pT3 N0 (0/25) L0 V0 Pn0 G2 R0, MSS, ECOG 0, Age 73 | 19 (18%) | 1 (14%) |
| Missing | 5 | 0 |
| Colon cancer UICC Stage II pT4a N0 (0/25) L0 V0 Pn0 G2 R0, MSS, ECOG 0, Age 68 | 68 (67%) | 5 (71%) |
| Missing | 7 | 0 |
| Rectal cancer 10 cm from the anal verge, pretherapeutic staging cT3 N+ M0 CRM- EMVI- after long-term neoadjuvant radiochemotherapy (ARO scheme 50.4 Gy with Capecitabine) and postoperative UICC Stage I (ypT2 N0 (0/25) L0 V0 Pn0 R0 CRM-, Regression grade II), ECOG 0, Age 68 | 64 (62%) | 3 (43%) |
| Missing | 4 | 0 |
| Rectal cancer 10 cm from the anal verge, pretherapeutic staging cT3 N0 M0 CRM- EMVI- after short-term radiotherapy (5x5 Gy) and postoperative UICC Stage III (ypT3 N1 (2/25) L0 V0 Pn0 R0, CRM- EMVI-), ECOG 0, Age 68 | 97 (94%) | 7 (100%) |
| Missing | 5 | 0 |
| Rectal cancer 10 cm from the anal verge, pretherapeutic staging cT1 N0 M0 CRM- EMVI- and postoperative Stage I (pT1, N0 L0 V0 R0, G2), ECOG 0, Age 68 | 1 (1.0%) | 0 (0%) |
| Missing | 4 | 0 |
| Colon cancer of the left flexure, postoperative Stage III (pT3, N2 (4/28) L0 V1 R0, G3), ECOG 0, Age 60 | 105 (100%) | 7 (100%) |
| Missing | 3 | 0 |

^a^ Funded by German Cancer Aid

**E**

| **Decision in favor of adjuvant chemotherapy in the case vignette,** **n (%)** | **Colorectal cancer center**, N = 58 | **Colorectal cancer center within an oncological center**, N = 57 |
| --- | --- | --- |
| Colon cancer UICC Stage III pT3 N1 (2/25) L0 V0 Pn0 R0, ECOG 0, Age 81 | 52 (91%) | 53 (93%) |
| Missing | 1 | 0 |
| Colon cancer UICC Stage II pT3 N0 (0/25) L0 V0 Pn0 G2 R0, MSS, ECOG 0, Age 73 | 11 (21%) | 9 (16%) |
| Missing | 5 | 0 |
| Colon cancer UICC Stage II pT4a N0 (0/25) L0 V0 Pn0 G2 R0, MSS, ECOG 0, Age 68 | 39 (74%) | 34 (62%) |
| Missing | 5 | 2 |
| Rectal cancer 10 cm from the anal verge, pretherapeutic staging cT3 N+ M0 CRM- EMVI- after long-term neoadjuvant radiochemotherapy (ARO scheme 50.4 Gy with Capecitabine) and postoperative UICC Stage I (ypT2 N0 (0/25) L0 V0 Pn0 R0 CRM-, Regression grade II), ECOG 0, Age 68 | 29 (54%) | 38 (67%) |
| Missing | 4 | 0 |
| Rectal cancer 10 cm from the anal verge, pretherapeutic staging cT3 N0 M0 CRM- EMVI- after short-term radiotherapy (5x5 Gy) and postoperative UICC Stage III (ypT3 N1 (2/25) L0 V0 Pn0 R0, CRM- EMVI-), ECOG 0, Age 68 | 47 (89%) | 57 (100%) |
| Missing | 5 | 0 |
| Rectal cancer 10 cm from the anal verge, pretherapeutic staging cT1 N0 M0 CRM- EMVI- and postoperative Stage I (pT1, N0 L0 V0 R0, G2), ECOG 0, Age 68 | 0 (0%) | 1 (1.8%) |
| Missing | 4 | 0 |
| Colon cancer of the left flexure, postoperative Stage III (pT3, N2 (4/28) L0 V1 R0, G3), ECOG 0, Age 60 | 55 (100%) | 57 (100%) |
| Missing | 3 | 0 |

**F**

| **Decision in favor of adjuvant chemotherapy in the case vignette,** **n (%)** | **< 80 cases of primary operative colorectal cancer**, N = 54 | **≥ 80 cases of primary operative colorectal cancer**, N = 55 |
| --- | --- | --- |
| Colon cancer UICC Stage III pT3 N1 (2/25) L0 V0 Pn0 R0, ECOG 0, Age 81 | 50 (93%) | 50 (93%) |
| Missing | 0 | 1 |
| Colon cancer UICC Stage II pT3 N0 (0/25) L0 V0 Pn0 G2 R0, MSS, ECOG 0, Age 73 | 12 (23%) | 7 (13%) |
| Missing | 1 | 0 |
| Colon cancer UICC Stage II pT4a N0 (0/25) L0 V0 Pn0 G2 R0, MSS, ECOG 0, Age 68 | 38 (70%) | 33 (63%) |
| Missing | 0 | 3 |
| Rectal cancer 10 cm from the anal verge, pretherapeutic staging cT3 N+ M0 CRM- EMVI- after long-term neoadjuvant radiochemotherapy (ARO scheme 50.4 Gy with Capecitabine) and postoperative UICC Stage I (ypT2 N0 (0/25) L0 V0 Pn0 R0 CRM-, Regression grade II), ECOG 0, Age 68 | 33 (61%) | 32 (58%) |
| Missing | 0 | 0 |
| Rectal cancer 10 cm from the anal verge, pretherapeutic staging cT3 N0 M0 CRM- EMVI- after short-term radiotherapy (5x5 Gy) and postoperative UICC Stage III (ypT3 N1 (2/25) L0 V0 Pn0 R0, CRM- EMVI-), ECOG 0, Age 68 | 50 (94%) | 53 (96%) |
| Missing | 1 | 0 |
| Rectal cancer 10 cm from the anal verge, pretherapeutic staging cT1 N0 M0 CRM- EMVI- and postoperative Stage I (pT1, N0 L0 V0 R0, G2), ECOG 0, Age 68 | 0 (0%) | 1 (1.8%) |
| Missing | 0 | 0 |
| Colon cancer of the left flexure, postoperative Stage III (pT3, N2 (4/28) L0 V1 R0, G3), ECOG 0, Age 60 | 54 (100%) | 55 (100%) |
| Missing | 0 | 0 |
