# Supplementary material for: Heterogeneity of treatment preferences in the absence of guideline recommendations – a case vignette study in colorectal cancer tumor boards in Germany, Austria and Switzerland
Source: BMC Gastroenterol. 2025 Oct 7;25:700. doi: 10.1186/s12876-025-04183-5 (PMC12505869; doi:10.1186/s12876-025-04183-5)
Supplement: Supplementary file 4 — Supplementary Material 4 [file 12876_2025_4183_MOESM4_ESM.docx]

**Supplement 4**

**A**


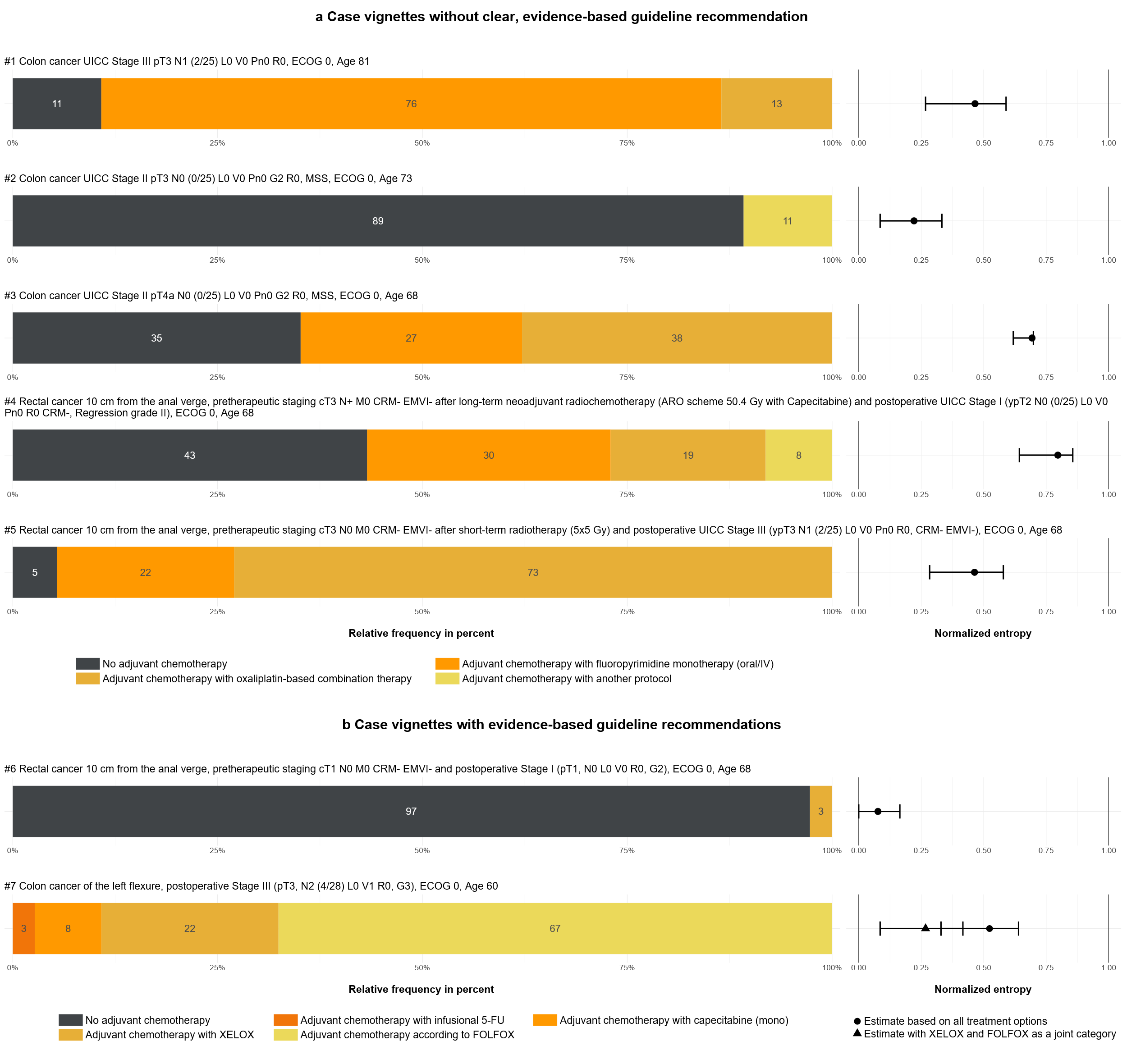


*Treatment preferences of 37 visceral oncological tumor boards*

*Note.* Frequency values in cases of unclear (a) and evidence based (b) guideline recommendation are reported within the bars in percent. Entropy estimates show the heterogeneity between 0 (Maximum agreement of responses) and 1 (Equal distribution across all 4 (#1–5) or 5 (#6–7) possible responses). In order to illustrate the heterogeneity with regard to the guideline recommendation 8.9., an additional estimate was given for vignette #7 in which the oxaliplatin-containing treatment options (XELOX and FLOFOX) were jointly categorized. Exact estimates and 95% confidence intervals are reported in Supplement 6B. N = 37.

**B**

*Heterogeneity of treatment preferences of 37 visceral oncological tumor boards*

| **Case number** | **Case description** | **Normalized entropy (95% CI)** |
| --- | --- | --- |
| #1 | Colon cancer UICC Stage III pT3 N1 (2/25) L0 V0 Pn0 R0, ECOG 0, Age 81 | 0.47 (0.27 to 0.59) |
| #2 | Colon cancer UICC Stage II pT3 N0 (0/25) L0 V0 Pn0 G2 R0, MSS, ECOG 0, Age 73 | 0.22 (0.09 to 0.33) |
| #3 | Colon cancer UICC Stage II pT4a N0 (0/25) L0 V0 Pn0 G2 R0, MSS, ECOG 0, Age 68 | 0.69 (0.62 to 0.70) |
| #4 | Rectal cancer 10 cm from the anal verge, pretherapeutic staging cT3 N+ M0 CRM- EMVI- after long-term neoadjuvant radiochemotherapy (ARO scheme 50.4 Gy with Capecitabine) and postoperative UICC Stage I (ypT2 N0 (0/25) L0 V0 Pn0 R0 CRM-, Regression grade II), ECOG 0, Age 68 | 0.80 (0.65 to 0.86) |
| #5 | Rectal cancer 10 cm from the anal verge, pretherapeutic staging cT3 N0 M0 CRM- EMVI- after short-term radiotherapy (5x5 Gy) and postoperative UICC Stage III (ypT3 N1 (2/25) L0 V0 Pn0 R0, CRM- EMVI-), ECOG 0, Age 68 | 0.46 (0.28 to 0.58) |
| #6 | Rectal cancer 10 cm from the anal verge, pretherapeutic staging cT1 N0 M0 CRM- EMVI- and postoperative Stage I (pT1, N0 L0 V0 R0, G2), ECOG 0, Age 68 | 0.08 (0.00 to 0.16) |
| #7 | Colon cancer of the left flexure, postoperative Stage III (pT3, N2 (4/28) L0 V1 R0, G3), ECOG 0, Age 60 | 0.52 (0.33 to 0.64) |
|  | XELOX and FOLFOX as a joint category | 0.31 (0.10 to 0.48) |

**C**


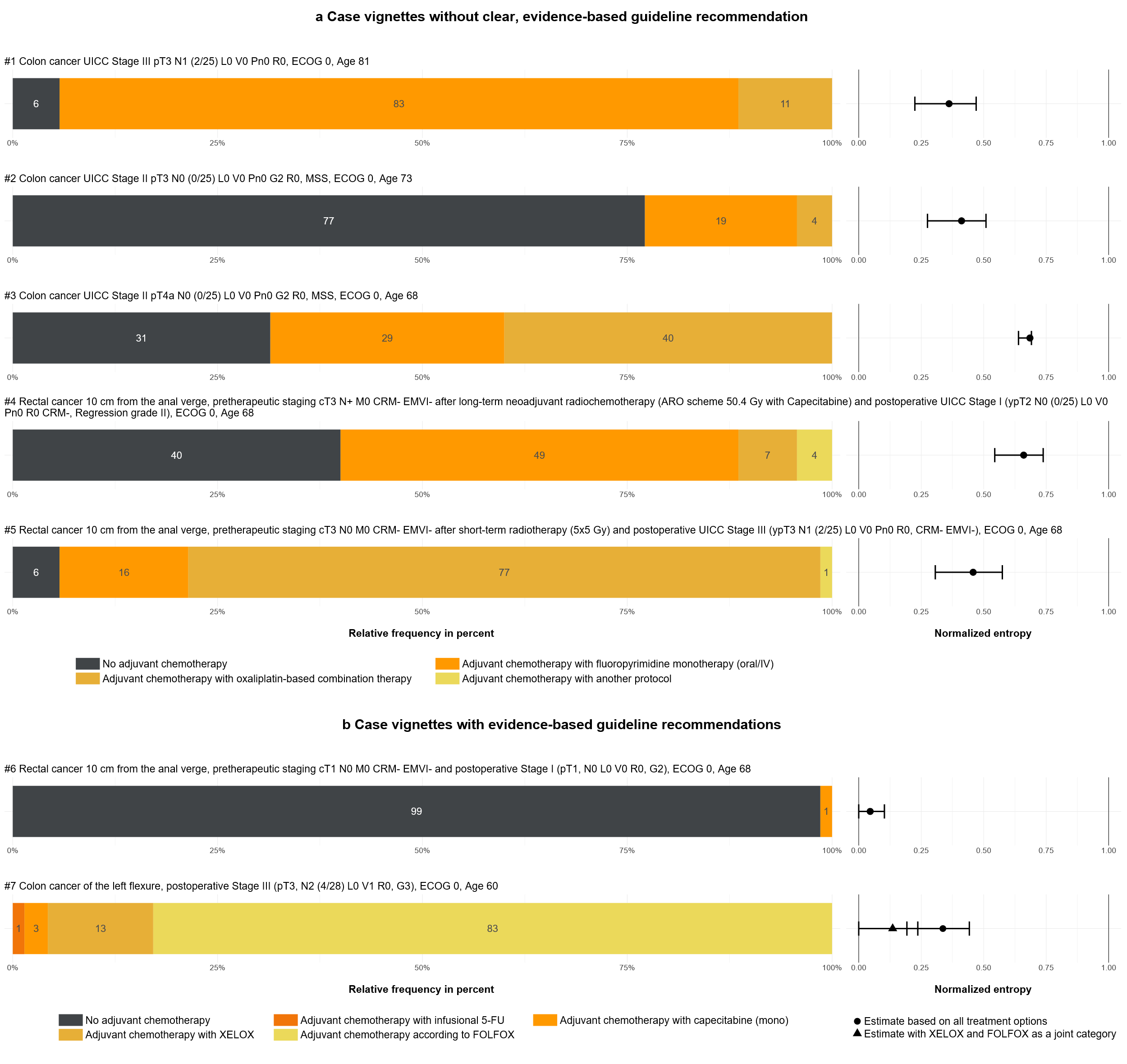


*Treatment preferences of 70 deputies of visceral oncological tumor boards*

*Note.* Frequency values in cases of unclear (a) and evidence based (b) guideline recommendation are reported within the bars in percent. Entropy estimates show the heterogeneity between 0 (Maximum agreement of responses) and 1 (Equal distribution across all 4 (#1–5) or 5 (#6–7) possible responses). In order to illustrate the heterogeneity with regard to the guideline recommendation 8.9., an additional estimate was given for vignette #7 in which the oxaliplatin-containing treatment options (XELOX and FLOFOX) were jointly categorized. Exact estimates and 95% confidence intervals are reported in Supplement 6D. N = 70.

**D**

*Heterogeneity of treatment preferences of 70 deputies of visceral oncological tumor boards*

| **Case number** | **Case description** | **Normalized entropy (95% CI)** |
| --- | --- | --- |
| #1 | Colon cancer UICC Stage III pT3 N1 (2/25) L0 V0 Pn0 R0, ECOG 0, Age 81 | 0.36 (0.22 to 0.47) |
| #2 | Colon cancer UICC Stage II pT3 N0 (0/25) L0 V0 Pn0 G2 R0, MSS, ECOG 0, Age 73 | 0.41 (0.28 to 0.51) |
| #3 | Colon cancer UICC Stage II pT4a N0 (0/25) L0 V0 Pn0 G2 R0, MSS, ECOG 0, Age 68 | 0.69 (0.64 to 0.69) |
| #4 | Rectal cancer 10 cm from the anal verge, pretherapeutic staging cT3 N+ M0 CRM- EMVI- after long-term neoadjuvant radiochemotherapy (ARO scheme 50.4 Gy with Capecitabine) and postoperative UICC Stage I (ypT2 N0 (0/25) L0 V0 Pn0 R0 CRM-, Regression grade II), ECOG 0, Age 68 | 0.66 (0.54 to 0.74) |
| #5 | Rectal cancer 10 cm from the anal verge, pretherapeutic staging cT3 N0 M0 CRM- EMVI- after short-term radiotherapy (5x5 Gy) and postoperative UICC Stage III (ypT3 N1 (2/25) L0 V0 Pn0 R0, CRM- EMVI-), ECOG 0, Age 68 | 0.46 (0.30 to 0.57) |
| #6 | Rectal cancer 10 cm from the anal verge, pretherapeutic staging cT1 N0 M0 CRM- EMVI- and postoperative Stage I (pT1, N0 L0 V0 R0, G2), ECOG 0, Age 68 | 0.05 (0.00 to 0.10) |
| #7 | Colon cancer of the left flexure, postoperative Stage III (pT3, N2 (4/28) L0 V1 R0, G3), ECOG 0, Age 60 | 0.34 (0.19 to 0.44) |
|  | XELOX and FOLFOX as a joint category | 0.16 (0.00 to 0.29) |

**E**


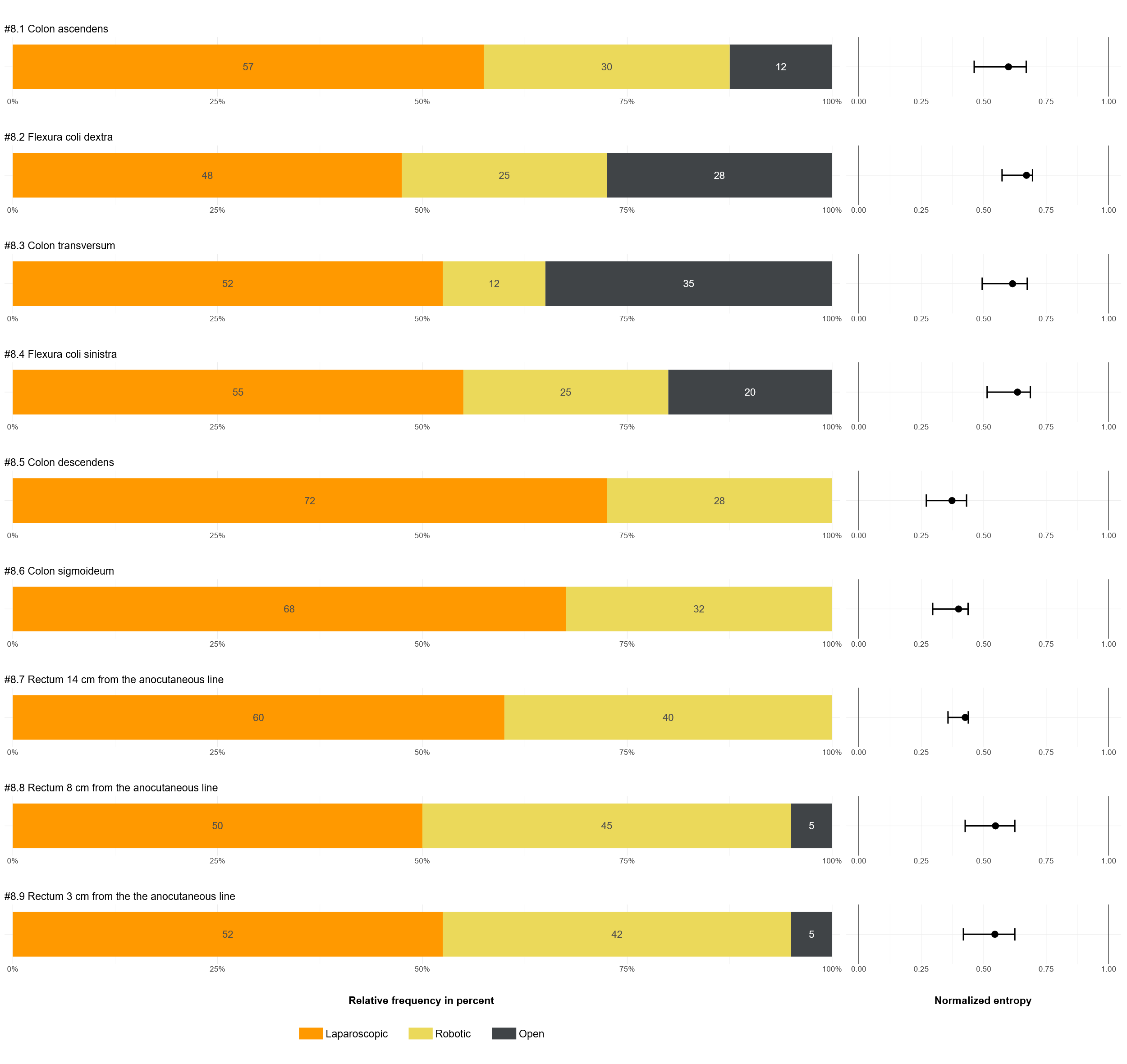


*Preferred resection technique of 40 visceral surgery departments*

*Note.* Frequency values of preferred resection technique for a patient (BMI 25, no previous surgery) with colorectal cancer cT3 N1 depending on the localization are reported within the bars in percent. Entropy estimates show the heterogeneity between 0 (Maximum agreement of responses) and 1 (Equal distribution across all 4 possible responses). Exact estimates and 95% confidence intervals are reported in Supplement 6F. N = 40.

**F**

*Heterogeneity of treatment preferences of 40 visceral surgery departments*

| **Case number** | **Case description** | **Normalized entropy (95% CI)** |
| --- | --- | --- |
| #8.1 | Colon ascendens | 0.60 (0.46 to 0.67) |
| #8.2 | Flexura coli dextra | 0.67 (0.57 to 0.70) |
| #8.3 | Colon transversum | 0.62 (0.49 to 0.68) |
| #8.4 | Flexura coli sinistra | 0.64 (0.50 to 0.69) |
| #8.5 | Colon descendens | 0.37 (0.27 to 0.43) |
| #8.6 | Colon sigmoideum | 0.40 (0.30 to 0.44) |
| #8.7 | Rectum 14 cm from the anocutaneous line | 0.43 (0.36 to 0.44) |
| #8.8 | Rectum 8 cm from the anocutaneous line | 0.55 (0.43 to 0.62) |
| #8.9 | Rectum 3 cm from the the anocutaneous line | 0.54 (0.42 to 0.62) |

**G**

*
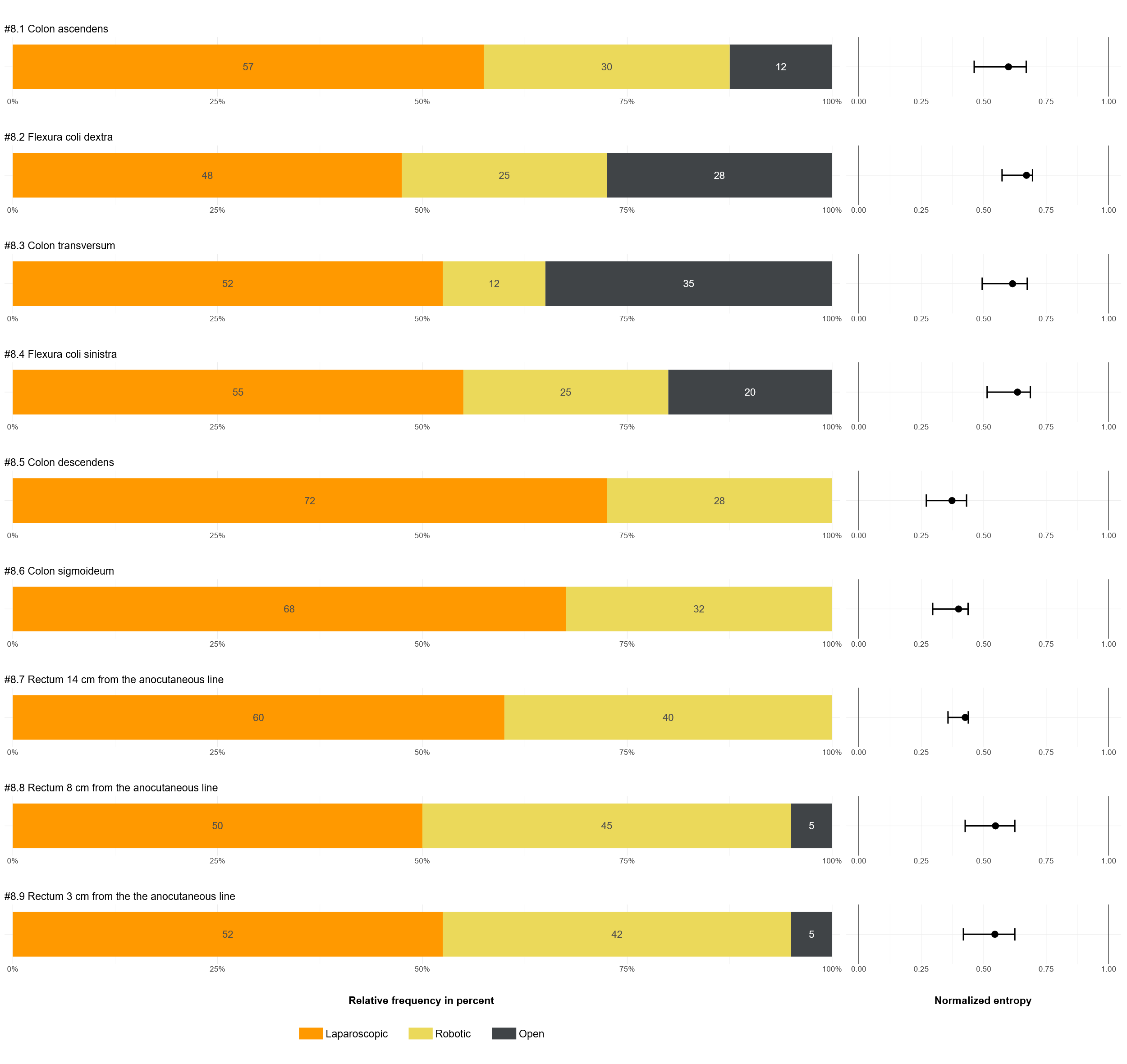
*

*Preferred resection technique of 71 deputies of the visceral surgery departments*

*Note.* Frequency values of preferred resection technique for a patient (BMI 25, no previous surgery) with colorectal cancer cT3 N1 depending on the localization are reported within the bars in percent. Entropy estimates show the heterogeneity between 0 (Maximum agreement of responses) and 1 (Equal distribution across all 4 possible responses). Exact estimates and 95% confidence intervals are reported in Supplement 6H. N = 71.

**H**

*Heterogeneity of treatment preferences of 71 deputies of the visceral surgery departments*

| **Case number** | **Case description** | **Normalized entropy (95% CI)** |
| --- | --- | --- |
| #8.1 | Colon ascendens | 0.66 (0.59 to 0.69) |
| #8.2 | Flexura coli dextra | 0.67 (0.62 to 0.69) |
| #8.3 | Colon transversum | 0.62 (0.54 to 0.67) |
| #8.4 | Flexura coli sinistra | 0.64 (0.56 to 0.68) |
| #8.5 | Colon descendens | 0.56 (0.45 to 0.63) |
| #8.6 | Colon sigmoideum | 0.48 (0.39 to 0.55) |
| #8.7 | Rectum 14 cm from the anocutaneous line | 0.51 (0.42 to 0.57) |
| #8.8 | Rectum 8 cm from the anocutaneous line | 0.51 (0.42 to 0.57) |
| #8.9 | Rectum 3 cm from the the anocutaneous line | 0.59 (0.48 to 0.67) |
