# Supplementary material for: Heterogeneity of treatment preferences in the absence of guideline recommendations – a case vignette study in colorectal cancer tumor boards in Germany, Austria and Switzerland
Source: BMC Gastroenterol. 2025 Oct 7;25:700. doi: 10.1186/s12876-025-04183-5 (PMC12505869; doi:10.1186/s12876-025-04183-5)
Supplement: Supplementary file 5 — Supplementary Material 5 [file 12876_2025_4183_MOESM5_ESM.docx]

**Supplement 5**

**a Observed combinations of preferred resection techniques for colon cancer in different locations**
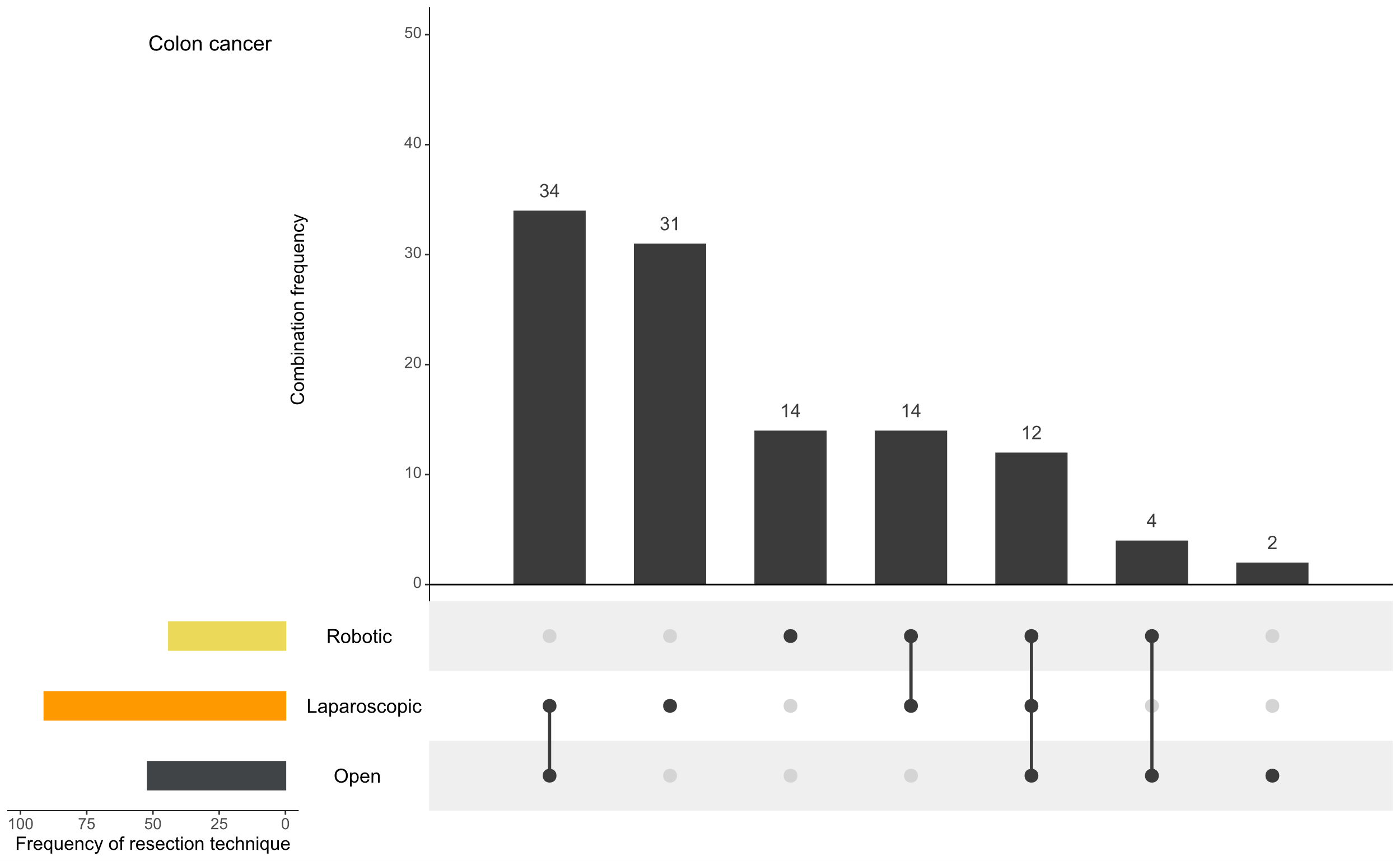


**b Observed combinations of preferred resection techniques for rectum cancer in different locations**
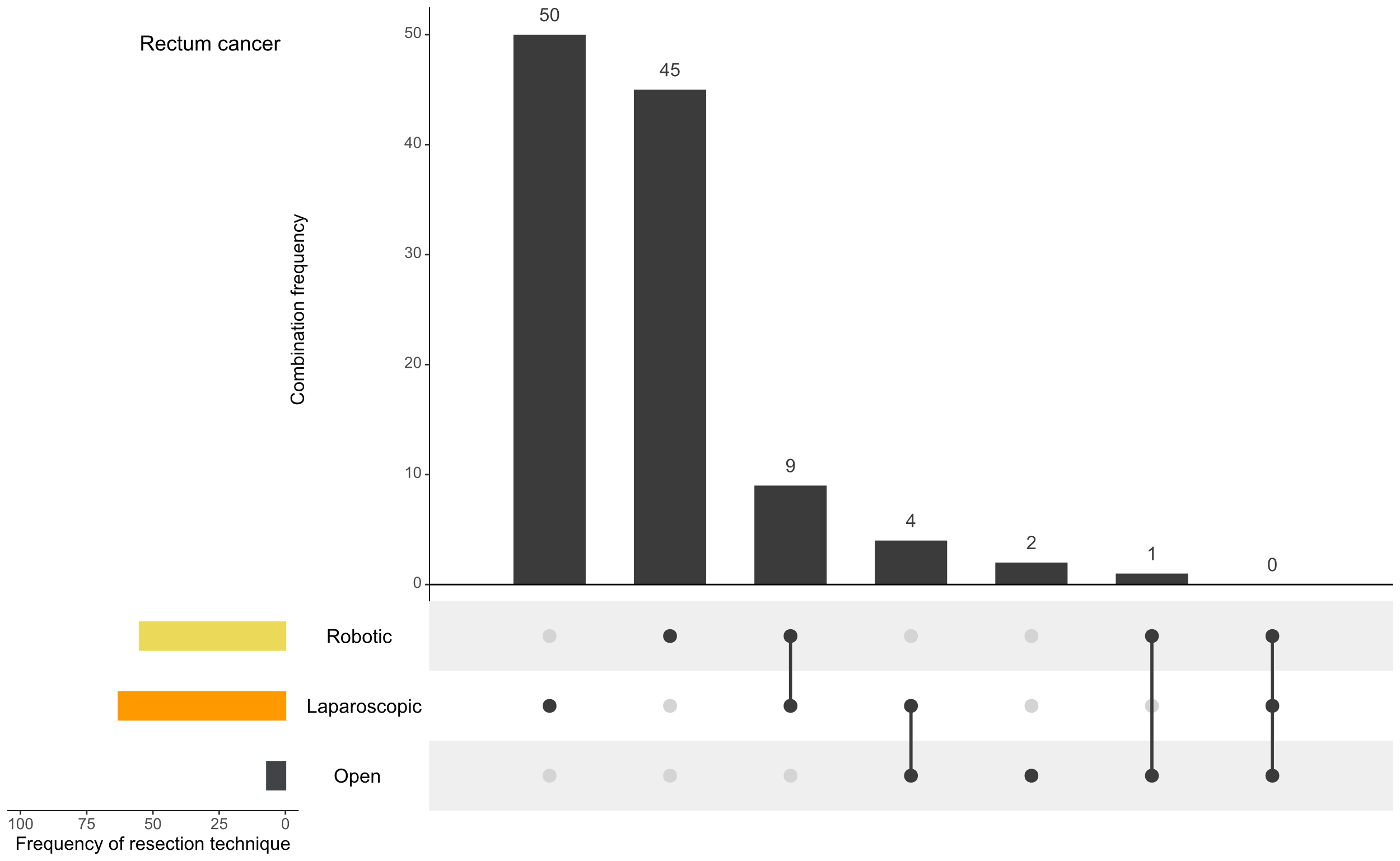


*Note.* Frequency values are reported in absolute numbers, N = 111

The UpSet Plots (matrix-based visualization) show the range of surgical access typically used within one center as well as the distribution of these combinations in the sample.
