# Supplementary material for: Heterogeneity of treatment preferences in the absence of guideline recommendations – a case vignette study in colorectal cancer tumor boards in Germany, Austria and Switzerland
Source: BMC Gastroenterol. 2025 Oct 7;25:700. doi: 10.1186/s12876-025-04183-5 (PMC12505869; doi:10.1186/s12876-025-04183-5)
Supplement: Supplementary file 6 — Supplementary Material 6 [file 12876_2025_4183_MOESM6_ESM.docx]

**Supplement 6**

**A**

| **Preference for minimally invasive surgery in the case vignette,** **n (%)** | **Actual response of a tumor board**, N = 42 | **Response of the center coordinator or deputy on behalf of the tumor board**, N = 73 |
| --- | --- | --- |
| Colon ascendens | 35 (88%) | 56 (78%) |
| Missing | 2 | 1 |
| Flexura coli dextra | 29 (73%) | 48 (68%) |
| Missing | 2 | 2 |
| Colon transversum | 26 (65%) | 35 (49%) |
| Missing | 2 | 1 |
| Flexura coli sinistra | 32 (80%) | 52 (72%) |
| Missing | 2 | 1 |
| Colon descendens | 40 (98%) | 64 (89%) |
| Missing | 1 | 1 |
| Colon sigmoideum | 40 (100%) | 70 (97%) |
| Missing | 2 | 1 |
| Rectum 14 cm from the anocutaneous line | 40 (100%) | 70 (96%) |
| Missing | 2 | 0 |
| Rectum 8 cm from the anocutaneous line | 38 (95%) | 70 (97%) |
| Missing | 2 | 1 |
| Rectum 3 cm from the the anocutaneous line | 39 (95%) | 66 (94%) |
| Missing | 1 | 1 |

**B**

| **Preference for minimally invasive surgery in the case vignette,** **n (%)** | **No University Hospital**, N = 99 | **University Hospital**, N = 14 |
| --- | --- | --- |
| Colon ascendens | 78 (80%) | 13 (93%) |
| Missing | 1 | 0 |
| Flexura coli dextra | 65 (67%) | 12 (86%) |
| Missing | 2 | 0 |
| Colon transversum | 52 (53%) | 9 (64%) |
| Missing | 1 | 0 |
| Flexura coli sinistra | 74 (76%) | 10 (71%) |
| Missing | 1 | 0 |
| Colon descendens | 93 (95%) | 11 (79%) |
| Missing | 1 | 0 |
| Colon sigmoideum | 96 (98%) | 14 (100%) |
| Missing | 1 | 0 |
| Rectum 14 cm from the anocutaneous line | 96 (97%) | 14 (100%) |
| Missing | 0 | 0 |
| Rectum 8 cm from the anocutaneous line | 95 (97%) | 13 (93%) |
| Missing | 1 | 0 |
| Rectum 3 cm from the the anocutaneous line | 92 (94%) | 14 (100%) |
| Missing | 1 | 0 |

**C**

| **Preference for minimally invasive surgery in the case vignette,** **n (%)** | **No teaching hospital**, N = 21 | **Teaching hospital**, N = 92 |
| --- | --- | --- |
| Colon ascendens | 18 (90%) | 73 (79%) |
| Missing | 1 | 0 |
| Flexura coli dextra | 15 (75%) | 62 (68%) |
| Missing | 1 | 1 |
| Colon transversum | 10 (50%) | 51 (55%) |
| Missing | 1 | 0 |
| Flexura coli sinistra | 13 (65%) | 71 (77%) |
| Missing | 1 | 0 |
| Colon descendens | 16 (80%) | 88 (96%) |
| Missing | 1 | 0 |
| Colon sigmoideum | 19 (95%) | 91 (99%) |
| Missing | 1 | 0 |
| Rectum 14 cm from the anocutaneous line | 19 (90%) | 91 (99%) |
| Missing | 0 | 0 |
| Rectum 8 cm from the anocutaneous line | 18 (90%) | 90 (98%) |
| Missing | 1 | 0 |
| Rectum 3 cm from the the anocutaneous line | 18 (90%) | 88 (96%) |
| Missing | 1 | 0 |

**D**

| **Preference for minimally invasive surgery in the case vignette,** **n (%)** | **No Comprehensive Cancer Center^a^,** N = 106 | **Comprehensive Cancer Center^a^**, N = 7 |
| --- | --- | --- |
| Colon ascendens | 84 (81%) | 6 (86%) |
| Missing | 1 | 0 |
| Flexura coli dextra | 71 (68%) | 6 (86%) |
| Missing | 2 | 0 |
| Colon transversum | 58 (55%) | 3 (43%) |
| Missing | 1 | 0 |
| Flexura coli sinistra | 80 (76%) | 4 (57%) |
| Missing | 1 | 0 |
| Colon descendens | 98 (94%) | 5 (71%) |
| Missing | 1 | 0 |
| Colon sigmoideum | 103 (98%) | 7 (100%) |
| Missing | 1 | 0 |
| Rectum 14 cm from the anocutaneous line | 103 (98%) | 7 (100%) |
| Missing | 0 | 0 |
| Rectum 8 cm from the anocutaneous line | 102 (97%) | 6 (86%) |
| Missing | 1 | 0 |
| Rectum 3 cm from the the anocutaneous line | 99 (94%) | 7 (100%) |
| Missing | 1 | 0 |

^a^ Funded by German Cancer Aid

**E**

| **Preference for minimally invasive surgery in the case vignette,** **n (%)** | **Colorectal cancer center**, N = 56 | **Colorectal cancer center within an oncological center**, N = 57 |
| --- | --- | --- |
| Colon ascendens | 45 (82%) | 46 (81%) |
| Missing | 1 | 0 |
| Flexura coli dextra | 40 (73%) | 37 (66%) |
| Missing | 1 | 1 |
| Colon transversum | 29 (53%) | 32 (56%) |
| Missing | 1 | 0 |
| Flexura coli sinistra | 37 (67%) | 47 (82%) |
| Missing | 1 | 0 |
| Colon descendens | 50 (91%) | 54 (95%) |
| Missing | 1 | 0 |
| Colon sigmoideum | 53 (96%) | 57 (100%) |
| Missing | 1 | 0 |
| Rectum 14 cm from the anocutaneous line | 53 (95%) | 57 (100%) |
| Missing | 0 | 0 |
| Rectum 8 cm from the anocutaneous line | 52 (95%) | 56 (98%) |
| Missing | 1 | 0 |
| Rectum 3 cm from the the anocutaneous line | 51 (93%) | 54 (96%) |
| Missing | 1 | 0 |

**F**

| **Preference for minimally invasive surgery in the case vignette,** **n (%)** | **< 80 cases of primary operative colorectal cancer**, N = 54 | **≥ 80 cases of primary operative colorectal cancer**, N = 55 |
| --- | --- | --- |
| Colon ascendens | 41 (76%) | 47 (85%) |
| Missing | 0 | 0 |
| Flexura coli dextra | 36 (67%) | 41 (76%) |
| Missing | 0 | 1 |
| Colon transversum | 31 (57%) | 30 (55%) |
| Missing | 0 | 0 |
| Flexura coli sinistra | 41 (76%) | 43 (78%) |
| Missing | 0 | 0 |
| Colon descendens | 50 (93%) | 51 (93%) |
| Missing | 0 | 0 |
| Colon sigmoideum | 52 (96%) | 55 (100%) |
| Missing | 0 | 0 |
| Rectum 14 cm from the anocutaneous line | 52 (96%) | 55 (100%) |
| Missing | 0 | 0 |
| Rectum 8 cm from the anocutaneous line | 51 (94%) | 54 (98%) |
| Missing | 0 | 0 |
| Rectum 3 cm from the the anocutaneous line | 49 (91%) | 54 (98%) |
| Missing | 0 | 0 |
