# Supplementary material for: Heterogeneity of treatment preferences in the absence of guideline recommendations – a case vignette study in colorectal cancer tumor boards in Germany, Austria and Switzerland
Source: BMC Gastroenterol. 2025 Oct 7;25:700. doi: 10.1186/s12876-025-04183-5 (PMC12505869; doi:10.1186/s12876-025-04183-5)
Supplement: Supplementary file 7 — Supplementary Material 7 [file 12876_2025_4183_MOESM7_ESM.docx]

**Supplement 7**

**A**

| **Case number** | **Case description** | **Normalized entropy (95% CI)** |
| --- | --- | --- |
| #1 | Colon cancer UICC Stage III pT3 N1 (2/25) L0 V0 Pn0 R0, ECOG 0, Age 81 | 0.39 (0.29 to 0.48) |
| #2 | Colon cancer UICC Stage II pT3 N0 (0/25) L0 V0 Pn0 G2 R0, MSS, ECOG 0, Age 73 | 0.41 (0.28 to 0.51) |
| #3 | Colon cancer UICC Stage II pT4a N0 (0/25) L0 V0 Pn0 G2 R0, MSS, ECOG 0, Age 68 | 0.68 (0.65 to 0.69) |
| #4 | Rectal cancer 10 cm from the anal verge, pretherapeutic staging cT3 N+ M0 CRM- EMVI- after long-term neoadjuvant radiochemotherapy (ARO scheme 50.4 Gy with Capecitabine) and postoperative UICC Stage I (ypT2 N0 (0/25) L0 V0 Pn0 R0 CRM-, Regression grade II), ECOG 0, Age 68 | 0.71 (0.63 to 0.77) |
| #5 | Rectal cancer 10 cm from the anal verge, pretherapeutic staging cT3 N0 M0 CRM- EMVI- after short-term radiotherapy (5x5 Gy) and postoperative UICC Stage III (ypT3 N1 (2/25) L0 V0 Pn0 R0, CRM- EMVI-), ECOG 0, Age 68 | 0.46 (0.34 to 0.55) |
| #6 | Rectal cancer 10 cm from the anal verge, pretherapeutic staging cT1 N0 M0 CRM- EMVI- and postoperative Stage I (pT1, N0 L0 V0 R0, G2), ECOG 0, Age 68 | 0.06 (0.00 to 0.13) |
| #7 | Colon cancer of the left flexure, postoperative Stage III (pT3, N2 (4/28) L0 V1 R0, G3), ECOG 0, Age 60 | 0.40 (0.29 to 0.49) |
|  | XELOX and FOLFOX as a joint category | 0.21 (0.08 to 0.31) |

**B**

| **Case number** | **Case description** | **Normalized entropy (95% CI)** |
| --- | --- | --- |
| #8.1 | Colon ascendens | 0.64 (0.58 to 0.67) |
| #8.2 | Flexura coli dextra | 0.67 (0.62 to 0.69) |
| #8.3 | Colon transversum | 0.62 (0.57 to 0.66) |
| #8.4 | Flexura coli sinistra | 0.64 (0.57 to 0.67) |
| #8.5 | Colon descendens | 0.51 (0.42 to 0.57) |
| #8.6 | Colon sigmoideum | 0.46 (0.38 to 0.51) |
| #8.7 | Rectum 14 cm from the anocutaneous line | 0.48 (0.42 to 0.53) |
| #8.8 | Rectum 8 cm from the anocutaneous line | 0.52 (0.46 to 0.57) |
| #8.9 | Rectum 3 cm from the the anocutaneous line | 0.57 (0.49 to 0.63) |
